# The genome-wide impact of trisomy 21 on DNA methylation and its implications for hematopoiesis

**DOI:** 10.1038/s41467-021-21064-z

**Published:** 2021-02-05

**Authors:** Ivo S. Muskens, Shaobo Li, Thomas Jackson, Natalina Elliot, Helen M. Hansen, Swe Swe Myint, Priyatama Pandey, Jeremy M. Schraw, Ritu Roy, Joaquin Anguiano, Katerina Goudevenou, Kimberly D. Siegmund, Philip J. Lupo, Marella F. T. R. de Bruijn, Kyle M. Walsh, Paresh Vyas, Xiaomei Ma, Anindita Roy, Irene Roberts, Joseph L. Wiemels, Adam J. de Smith

**Affiliations:** 1 Center for Genetic Epidemiology, Department of Preventive Medicine, Keck School of Medicine of the University of Southern California, Los Angeles, CA USA; 2 Norris Comprehensive Cancer Center, University of Southern California, Los Angeles, USA; 3 Department of Paediatrics and MRC Molecular Haematology Unit, Weatherall Institute of Molecular Medicine, Oxford University and BRC Blood Theme, NIHR Oxford Biomedical Centre, Oxford, UK; 4 Department of Neurological Surgery, University of California San Francisco, San Francisco, CA USA; 5 Department of Pediatrics, Section of Hematology-Oncology, Baylor College of Medicine, Houston, TX USA; 6 Texas Children’s Cancer and Hematology Centers, Texas Children’s Hospital, Houston, TX USA; 7 Computational Biology and Informatics, University of California San Francisco, San Francisco, CA USA; 8 Department of Preventive Medicine, Keck School of Medicine of the University of Southern California, Los Angeles, CA USA; 9 MRC Molecular Haematology Unit, MRC Weatherall Institute of Molecular Medicine, University of Oxford, Oxford, UK; 10 Department of Neurosurgery, Duke University, Durham, NC USA; 11 Department of Pediatrics, Duke University, Durham, NC USA; 12 Department of Chronic Disease Epidemiology, Yale School of Public Health, New Haven, CT USA

**Keywords:** Methylation analysis, Haematopoiesis, DNA methylation, Epidemiology

## Abstract

Down syndrome is associated with genome-wide perturbation of gene expression, which may be mediated by epigenetic changes. We perform an epigenome-wide association study on neonatal bloodspots comparing 196 newborns with Down syndrome and 439 newborns without Down syndrome, adjusting for cell-type heterogeneity, which identifies 652 epigenome-wide significant CpGs (*P* < 7.67 × 10^−8^) and 1,052 differentially methylated regions. Differential methylation at promoter/enhancer regions correlates with gene expression changes in Down syndrome versus non-Down syndrome fetal liver hematopoietic stem/progenitor cells (*P* < 0.0001). The top two differentially methylated regions overlap *RUNX1* and *FLI1*, both important regulators of megakaryopoiesis and hematopoietic development, with significant hypermethylation at promoter regions of these two genes. Excluding Down syndrome newborns harboring preleukemic *GATA1* mutations (*N* = 30), identified by targeted sequencing, has minimal impact on the epigenome-wide association study results. Down syndrome has profound, genome-wide effects on DNA methylation in hematopoietic cells in early life, which may contribute to the high frequency of hematological problems, including leukemia, in children with Down syndrome.

## Abstract (web summary)

Down syndrome has a high co-morbidity with immune and hematopoietic disorders. Here, the authors perform an epigenome-wide association study in newborns with and without Down syndrome to find differential methylation across the genome, including in hematopoietic regulators *RUNX1* and *FLI1*.

## Introduction

Down syndrome (DS), caused by constitutive trisomy of chromosome 21 (T21), is one of the most common genetic disorders^1^, and is associated with a spectrum of adverse phenotypes^2^. DS is characterized by defects in immune system development and in hematopoiesis, with DS fetuses having perturbed megakaryocyte/red cell and B-lymphoid development^3^ and DS children having a higher frequency of lymphopenia^4^ and infections^5^. Furthermore, children with DS have a 20–30-fold increased risk of acute lymphoblastic leukemia (ALL) and a 500-fold increased risk of acute megakaryoblastic leukemia (AMKL), while displaying a decreased risk of common adult-onset solid tumors^6,7^. Approximately 10% of DS newborns present with transient abnormal myelopoiesis (TAM), a preleukemic disorder associated with increased peripheral blood blast cells and pathognomonic somatic mutations in the X-linked erythro-megakaryocytic transcription factor (TF) gene *GATA1*^8^. A further 15–20% have acquired *GATA1* mutations without clinical features, so-called “Silent TAM^8^.” TAM and Silent TAM resolve spontaneously in most cases, but up to 20% acquire additional oncogenic mutations and develop frank AMKL^9,10^.

DS-related phenotypes vary greatly in presentation and penetrance^2,11^, and understanding the biological basis of that variation may highlight novel therapeutic approaches, and shed light on the etiology of these conditions in non-DS individuals^11^. Altered expression of genes, both on Hsa21 and genome-wide, is widely accepted to play a key role in the manifestation of DS-related phenotypes, many of which originate prenatally^3^. Several studies, including in monozygotic twins discordant for T21, provide strong evidence for the effect of T21 on the human transcriptome^12,13^, with substantial interindividual variability in expression patterns^14^.

Studying baseline epigenetic effects of T21 at birth is a powerful approach to pinpoint broad epigenetic landscapes and/or individual genes that underlie DS-related phenotypes. Nevertheless, comprehensive analysis of genome-wide DNA methylation changes in DS is lacking; previous studies comprised very few (*N* < 30) individuals, did not explore interethnic differences, nor account for the potential impact of somatic *GATA1* mutation-harboring clones^15–19^.

Here, we investigate T21-associated changes in DNA methylation among 196 DS and 439 non-DS newborn blood samples, and consider the potential confounding effects of somatic *GATA1* mutations in DS newborns assessed by targeted sequencing. Our epigenome-wide association study (EWAS) of DS identifies 652 significant CpGs and 1052 differentially methylated regions (DMRs) associated with DS, including significant hypermethylation at promoter regions of *RUNX1* and *FLI1*, both critical regulators of hematopoiesis. Further, we find that differential methylation at regulatory regions in newborns with DS correlates with gene expression patterns in DS fetal liver (FL) hematopoietic stem and progenitor cells (HSPC). This is the first multiethnic study of its kind and the largest epigenome-wide analysis of DS patients to-date, revealing insights into the etiology of DS-related phenotypes.

## Results

High-quality genome-wide DNA methylation data were obtained for 196 DS and 439 non-DS newborns using Illumina Infinium MethylationEPIC Beadchip genome-wide arrays, including 651,772 CpGs on autosomes in our analyses, with an average 99.9% CpGs with a detection *P* value < 0.01. Genome-wide copy-number analysis confirmed T21 in all DS newborns (Supplementary Fig. 1) and euploidy in all but one non-DS individual, who was excluded from subsequent analyses. Study characteristics of the 635 newborns (*N* = 357 Latinos, 178 non-Latino whites, 55 Asians, 34 non-Latino blacks, and 11 other) are presented in Table 1. DS newborns had a slightly lower mean gestational age at birth (*P* = 0.04) and birth weight (*P* = 0.001) than non-DS newborns, and a higher frequency of DS newborns were preterm (*P* = 0.0004) and/or small-for-gestational age (*P* < 0.0001) than non-DS newborns (Table 1). Age at sampling was higher in DS newborns, the majority being sampled on day 3 of life compared to day 2 for non-DS neonates (*P* < 0.0001).

**Table 1:** Baseline characteristics of newborns with and without Down syndrome included in our epigenome-wide association study.

	Non-DS (*N* = 439) *N* (%)	DS (*N* = 196) *N* (%)	*P* value
Sex			
Female	182 (41.5%)	106 (54.1%)	0.0034^a^
Male	257 (58.5%)	90 (45.9%)	
Race/ethnicity			
Asian	38 (8.7%)	17 (8.7%)	0.00044^a^
Latino	253 (57.6%)	104 (53.1%)	
Non-Latino white	124 (28.2%)	54 (27.6%)	
Non-Latino black	13 (3.0%)	21 (10.7%)	
Other	11 (2.5%)	0 (0%)	
Blood collection age (days)			
Mean (SD)	1.33 (±0.70)	2.49 (±2.04)	<0.0001^b^
Median (range)	1.13 (0–5.25)	1.75 (0–15.3)	
Missing	3 (0.7%)	5 (2.6%)	
Gestational age (weeks)			
Mean (SD)	39.2 (±2.0)	38.2 (±2.2)	0.041^b^
Median (range)	39.4 (26.4–44.7)	38.3 (26.4–44.7)	
Preterm (<37 weeks)	44 (10.6%)	39 (22.0%)	0.0004^a^
Missing	23 (5.2%)	19 (9.7%)	
Birth weight (kg)			
Mean (SD)	3.39 (±0.55)	3.01 (±0.73)	0.001^b^
Median (range)	3.41 (1.04–5.05)	3.01 (0.96–8.65)	
Small-for-gestational age^c^	24 (6.1%)	33 (19.3%)	<0.0001^a^
Missing	0 (0%)	6 (3.1%)	
Birth year			
Median (range)	2004 (2000–2008)	1998 (1996–1999)	<0.0001^d^
Missing	3 (0.7%)	2 (1.0%)	

*kg* kilogram, *SD* standard deviation, *DS* Down syndrome.

^a^*P* values calculated by two-sided Fisher’s exact test.

^b^*P* values calculated by linear regression with the birth-related variable as the dependent variable, DS status as the independent variable, and adjusting for the remaining birth-related variables, sex, plate, and race/ethnicity.

^c^Small-for-gestational age calculated according to the sex- and gestational age-based intrauterine growth curves previously developed using US data^85^. Note we were not able to calculate this for newborns born >42 weeks due to limitations of the reference data.

^d^*P* value calculated by the two-sided Wilcoxon rank-sum test.

### DNA methylation-based clustering separates DS and non-DS newborns

**Fig. 1:**
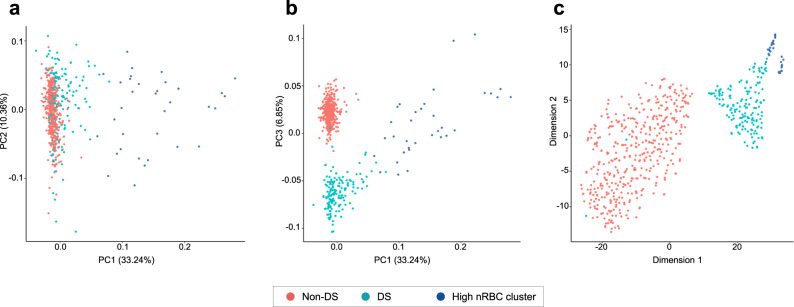
Principal components analysis (PCA) and t-distributed stochastic neighbor embedding (t-SNE) plots of blood-based DNA methylation data in Down syndrome (DS) and non-DS newborns. PCA and t-SNE plots were generated in R, using “prcomp” and “Rtsne” functions, respectively, using genome-wide DNA methylation data from Illumina Infinium EPICmethylation Beadchip arrays in 196 DS (teal/blue) and 439 non-DS (red) newborns, excluding CpG probes on chromosomes X, Y, and 21. The first three principal components (PC1, PC2, and PC3) explained 33.2%, 10.4%, and 6.9% of the variance, respectively. **a** Per-sample data for PC1 plotted against PC2. **b** PC1 versus PC3. DS newborns with high PC1 values also had high proportions of nucleated red blood cells (nRBCs) in deconvolution analyses; this cluster of 34 DS newborns is highlighted by the blue-colored circles in both plots. PC3 appears to be related to trisomy 21 status. **c** First and second t-SNE dimensions for DS (teal/blue) and non-DS (red) newborns. The cluster of 34 DS newborns with high nRBC proportions is highlighted by the blue-colored circles. Two DS newborns clustered with non-DS newborns in the PCA and t-SNE plots, and these were subsequently found to be likely mosaic for trisomy 21 (see Supplementary Fig. 2).

**Fig. 2:**
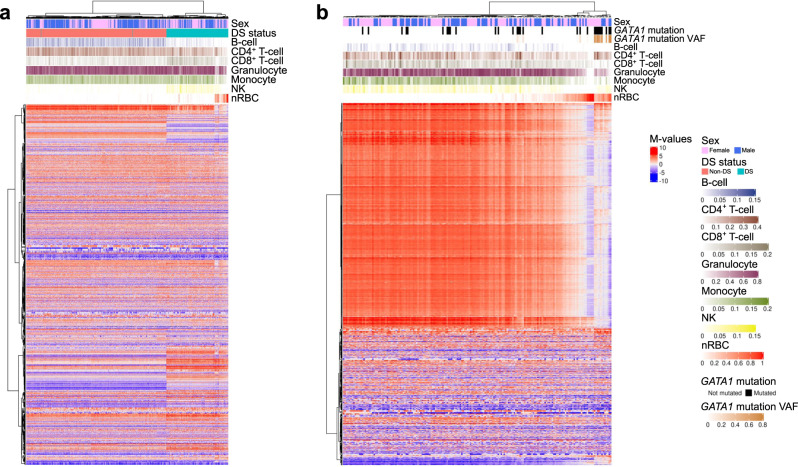
Heatmap and unsupervised hierarchical clustering of the top 2000 most variable CpGs genome-wide in Down syndrome (DS) and non-DS newborns, and by *GATA1* mutation status. These plots were generated using genome-wide DNA methylation data excluding chromosomes X, Y, and 21, and with *β* values converted to *M* values, using the “ComplexHeatmap” R package. Only the top 2000 CpGs with the greatest mean absolute deviation are displayed. **a** Hierarchical clustering and heatmap of 196 DS and 439 non-DS newborns, in relation to subject sex and deconvoluted blood cell proportions of B cells, CD4^+^ T cells, CD8^+^ T cells, granulocytes, monocytes, natural killer (NK) cells, and nucleated red blood cells (nRBCs). The first separation in hierarchical clustering represented DS (teal) versus non-DS (red) newborns. Two DS newborns clustered with the non-DS newborns, and were the same as those that clustered similarly in the t-SNE plot in Fig. 1. The DS newborns clustered into two main subgroups, those with high nRBC proportions and those with low nRBC proportions. **b** Hierarchical clustering and heatmap of 184 DS newborns, including 30 found to harbor somatic *GATA1* mutations via targeted sequencing, and 154 found to be *GATA1* mutation wildtype. DS newborns with *GATA1* mutations are represented by black bars, and the variant allele frequency (VAF) range of mutations shown in the row below. The first split appeared to separate DS newborns with high nRBC proportions from those with low nRBC proportions, whereas the second split was largely driven by subjects with *GATA1* mutations with high VAF. DS newborns with *GATA1* mutations with high VAF clustered together and tended to have high nRBC proportions; however, a proportion of *GATA1* wild-type newborns were also found to have high nRBC proportions, and these also clustered separately.

Visualization of principal components analysis (PCA) and t-distributed stochastic neighbor embedding (t-SNE) plots generated from genome-wide DNA methylation data, excluding CpG probes on sex chromosomes and Hsa21 and CpGs overlapping single-nucleotide polymorphisms (SNPs) with minor allele frequency (MAF) > 0.05, revealed clear separation of DS and non-DS newborns (Fig. 1). Intriguingly, a subset of 34 DS newborns departed from the DS cluster in the t-SNE plot, and the first PC (explaining 33.2% of overall variance) also stratified these DS newborns from the remainder. Unsupervised hierarchical clustering of the top 2000 most variable CpGs genome-wide (excluding chromosomes 21, X, and Y) resulted in similar grouping of subjects, with the first branch split separating DS from non-DS newborns, and the second split separating the same subset of 34 DS newborns among DS (Fig. 2a). Differences in blood cell proportions inferred from genome-wide DNA methylation data were seen between the three groups, as described in detail below. Two DS newborns clustered with non-DS newborns (Figs. 1 and 2) and visual inspection of copy-number plots revealed that both were likely mosaic for T21 (Supplementary Fig. 2).

### Targeted sequencing confirms high frequency of acquired *GATA1* variants in DS neonates

We detected 34 somatic *GATA1* mutations in 30 out of 184 (16.3%) DS newborns assessed by targeted sequencing (Supplementary Data 1). The 34 mutations displayed a wide range of variant allele frequencies (VAF: 0.96–96.1%). The mean VAF of predicted functional mutations (26.3%) was significantly higher than that of nonfunctional (noncoding/synonymous) somatic *GATA1* variants (VAF = 1.7%, *P* = 0.0034) (Supplementary Fig. 3). There was no significant association between the presence/absence or VAF of *GATA1* mutations and sex, race/ethnicity, birth weight, gestational age, or age at blood collection (Supplementary Table 1). In the hierarchical clustering, DS newborns with *GATA1* mutations with higher VAFs clustered together (Fig. 2b).

### Deconvolution of blood cell proportions

Next, we used reference-based cell-type deconvolution to address whether differences in DNA methylation between DS and non-DS neonates might reflect, or be confounded by, differences in the peripheral blood cellular composition. This confirmed several of the previously reported differences in neonatal blood cell proportions in DS compared with non-DS fetuses and newborns^8,20^ (Fig. 2a and Supplementary Fig. 4a), with similar patterns in Latino and non-Latino white newborns (Supplementary Fig. 5), including higher proportions of erythroblasts (nucleated red blood cells (nRBCs)) (*P* = 4.45 × 10^−65^) and lower proportions of B lymphocytes (*P* = 3.48 × 10^−28^) and T lymphocytes (CD4^+^ T lymphocytes) (*P* = 2.26 × 10^−53^) (Supplementary Fig. 4a and Supplementary Table 2)^8,20^. As the most dramatic difference was a subpopulation of DS newborns (*N* = 34, 17.3%) with a high proportion of erythroblasts (>25%), which clustered separately in Figs. 1 and 2, we next considered whether this identified the neonates with *GATA1* mutations. However, although we found a higher frequency of *GATA1* mutations in the newborns with increased erythroblasts compared to those with normal erythroblasts (12/33, 36.4% versus 18/151, 11.9%, *P* = 0.0015), the separate clustering of these cases is not primarily due to their *GATA1* mutation status (Supplementary Figs. 4b, 6 and Supplementary Table 2). Interestingly, deconvolution analysis suggested lower proportions of monocytes (*P* = 3.75 × 10^−23^) and granulocytes (*P* = 8.10 × 10^−32^) in DS neonates. As previous studies show increased monocytes and granulocytes in DS newborns, this likely reflects the limitations of deconvolution analysis where atypical cells are present, such as blast cells and dysplastic cells, which are common in DS neonates^8^, and lack a suitable reference “methylome” library. Taken together, the differences in peripheral blood cell composition support the use of robust adjustment for cell-type heterogeneity in our EWAS of DS. Rather than adjusting for the blood cell proportions estimated from our reference-based deconvolution described above, we opted to include components calculated using the reference-free, sparse PCA algorithm ReFACTor (see “Methods”) as covariates in our EWAS models.

### Epigenome-wide significant CpGs associated with DS

**Fig. 3:**
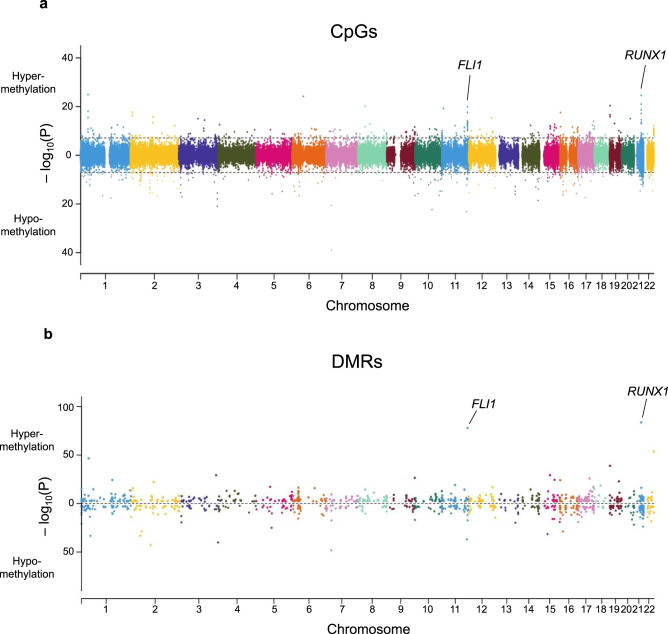
Bidirectional Manhattan plots displaying Down syndrome (DS)-associated CpGs and differentially methylated regions (DMRs) genome-wide. **a** Manhattan plot presenting the −log_10_(*P*) values of autosomal CpGs derived from the multiethnic epigenome-wide association study (EWAS) of DS. *P* values were calculated using linear regression testing the association of each CpG beta-value with DS, adjusting for sex, plate, the first ten ReFACToR principal components (PCs), and the first ten EPISTRUCTURE PCs. Points above zero correspond to CpGs that were hypermethylated in DS (*N* = 196) versus non-DS (*N* = 439) newborns, whereas points below zero correspond to hypomethylated CpGs in DS. The genomic inflation factor (*λ*) was 1.47. Dotted lines correspond to the threshold for epigenome-wide significance (*P* = 7.67 × 10^−8^) after Bonferroni correction for multiple testing. The strong association peaks at *FLI1* on chromosome 11 and *RUNX1* on chromosome 21 are highlighted. **b** Manhattan plot showing DMRs identified in the overall multiethnic analysis using DMRcate and comb-p. The –log_10_(*P*) values were derived from the Šidák-corrected *P* values from the more stringent method, comb-p, with the DS EWAS *P* values at each CpG as input. Points above zero correspond to DMRs with a mean Δ*β-*value above 0 (hypermethylated in DS), whereas points below zero correspond to hypomethylated DMRs in DS. The top two significant DMRs in *RUNX1* and *FLI1* are highlighted. We identified DMRs in several additional genes that regulate hematopoiesis and/or are known drivers of leukemogenesis, including at Hsa21 genes *DYRK1A* (DMR *N* = 3), *ERG* (*N* = 2), and *ETS2* (*N* = 1), and at non-Hsa21 genes *ETV6* (*N* = 3), *BCR* and *KIT* (both *N* = 2), and *GATA2*, *TET1*, *TET2*, *FLT3*, *DNMT3A*, *KAT6B*, *KMT2C*, *PBX1*, and *RARA* (all *N* = 1) (Supplementary Data 7).

To investigate the biological significance of the epigenome-wide changes in DS neonatal blood cells, we next assessed differential methylation of CpGs on autosomal chromosomes, including Hsa21, adjusted for variation in cell-type proportions, sex, and ancestry-informative PCs. A total of 652 DS-associated CpGs were detected following Bonferroni correction (*P* < 7.67 × 10^−8^, Fig. 3a and Supplementary Data 2), with 319/652 (48.9%) CpGs hypermethylated and 333/652 (51.1%) hypomethylated in DS compared with non-DS newborns. The pattern of DNA methylation was distinctly different for CpGs on Hsa21; the majority of significantly differentially methylated CpGs (64/79, 81.0%) were hypomethylated, whereas on other chromosomes, the proportions of hypomethylated CpGs were on average much lower (median: 43.5%, range: 16.0–80.0%). In addition, when considering all CpGs included in the EWAS, a significantly higher proportion of probes on Hsa21 were hypomethylated (4425/7351, 60.2%) compared with probes on all other autosomes combined (356,222/644,421, 55.3%, *P* < 0.0001, Chi-squared test), which was particularly the case in shores and shelves but not in CpG islands themselves (Supplementary Table 3).

Remarkably, 13 of 15 hypermethylated and epigenome-wide significant Hsa21 CpGs overlapped *RUNX1*, at the proximal P2 promoter (Supplementary Fig. 7). *RUNX1* was also identified when we considered the epigenome-wide significant CpGs that overlapped known genes (*N* = 357); of the top 20 significant CpGs, overlapping 11 unique genes, the three with the largest methylation differences (beta coefficients >0.38) were all located in *RUNX1* (Table 2 and Supplementary Data 2). The other ten genes included the megakaryocytic gene *FLI*, *SH3D21*, and *KIAA0087* (each with two CpGs), and *DST*, *VSIG2*, *KLF16*, *OLFML1*, *SETD3, CELF3*, and *NOL10* (one CpG). Of the four intergenic CpGs, two overlap a putative enhancer of *HES1* (Table 2)^21^. We repeated linear regression analyses for the top significant CpGs in *RUNX1* (cg12477880) and *FLI1* (cg17239923), genes that are known regulators of hematopoiesis, adjusting for deconvoluted blood cell proportions instead of ReFACTor components (see “Methods”), and the associations with DS remained highly significant (both *P* < 2.0 × 10^−16^).

**Table 2:** Top differentially methylated CpG probes associated with Down syndrome.

Chr	Position (hg19)	Gene	Probe	Beta coefficient^a^	*P* value^a^	CpG island overlap
7	26577897	*KIAA0087* (lncRNA)	cg07741821	−0.289	1.26 x 10^−39^	
1	36786777	*SH3D21*	cg02993069	0.198	1.14 x 10^−25^	Yes
21	36259241	*RUNX1*	cg12477880	0.383	2.32 x 10^−25^	Yes
6	56607099	*DST*	cg08882472	0.157	6.14 x 10^−25^	
11	124621829	*VSIG2*	cg24942416	−0.175	7.33 x 10^−24^	
10	85363826	Intergenic	cg07841633	−0.329	4.21 x 10^−23^	
21	36259383	*RUNX1*	cg00994804	0.388	9.36 x 10^−22^	Yes
3	193988737	Intergenic (*HES1* enhancer)	cg11218872	−0.121	1.05 x 10^−21^	
7	26578098	*KIAA0087* (lncRNA)	cg02451831	−0.159	2.67 x 10^−21^	
19	1851882	*KLF16*	cg13382072	0.180	4.07 x 10^−21^	Yes
8	37575051	Intergenic	cg24020235	0.188	7.30 x 10^−21^	
11	128556611	*FLI1*	cg17239923	0.232	1.21 x 10^−20^	
11	7519636	*OLFML1*	cg19030331	0.165	6.05 x 10^−20^	
21	36258497	*RUNX1*	cg03142697	0.396	2.15 x 10^−19^	
14	99880641	*SETD3*	cg24999883	−0.067	2.85 x 10^−19^	
1	36786615	*SH3D21*	cg12679760	0.183	9.10 x 10^−19^	Yes
3	193988507	Intergenic (*HES1* enhancer)	cg23719650	−0.107	1.11 x 10^−18^	
2	10830636	*NOL10*	cg11972401	0.058	2.10 x 10^−18^	
1	151672762	*CELF3*	cg23565347	−0.092	2.15 x 10^−18^	
11	128556341	*FLI1*	cg19765472	0.207	2.42 x 10^−18^	

^a^*P* values (not adjusted for multiple comparisons) and beta coefficients calculated in the multiethnic EWAS of Down syndrome, using linear regression adjusting for sex, plate, the first ten ReFACToR principal components (PCs), and the first ten EPISTRUCTURE PCs.

Neither removal of DS newborns with *GATA1* mutations (*N* = 30) nor those with high erythroblasts (*N* = 34) affected EWAS results substantially, and ethnicity-stratified analyses showed similar results, with 622/652 (95.4%) epigenome-wide significant CpGs showing the same direction of effect in both Latinos and non-Latino whites (Supplementary Data 2). In addition, in a subset of newborns with available birth weight and gestational age information (176 DS and 416 non-DS), we repeated the EWAS adjusting for these birth variables, but again, the results were not substantially altered (Supplementary Data 2). In a sex-stratified EWAS, no chromosome X CpGs were epigenome-wide significant in females, and 2 significant CpGs in males did not replicate in females (Supplementary Data 2).

We also determined the overlap of DS-associated CpGs with genomic locations and functional elements. Hypomethylated CpGs were significantly underrepresented at gene promoters and CpG islands (Supplementary Fig. 8 and Supplementary Data 3). Hypermethylated CpGs were significantly enriched at binding sites for *NFE2*, *MAFF*, *MAFK*, and *BACH1*, TFs that form components of a key erythro-megakaryocyte regulatory network (Supplementary Data 3). Hypermethylated CpGs were also significantly enriched at DNase I hypersensitive sites (DHS) (*P* = 1.67 × 10^−16^), at H3K4me1- and H3K4me3-binding sites in hematopoietic stem cells (HSCs, both *P* < 5.73 × 10^−10^), and at enhancer loci in HSCs (*P* < 3.27 × 10^−6^), whereas hypomethylated CpGs were significantly enriched at H3K36me3 sites (*P* = 9.92 × 10^−11^) (Supplementary Data 3), altogether indicating repression of gene expression.

Pathway analysis of genes overlapped by epigenome-wide significant CpGs revealed significant enrichment for 62 Gene Ontology (GO) terms, the majority related to hematopoiesis and immune function, and 12 Kyoto Encyclopedia of Genes and Genomes (KEGG) pathways, with hematopoietic cell lineage displaying the strongest enrichment in addition to several immune-related pathways (Supplementary Data 4).

We next assessed whether previously reported DS-associated CpGs were replicated. In two previous studies, from Bacalini et al. and Henneman et al.^16,17^, there were 111 DS-associated CpGs with concordant directions of effect. Of these, 97 were present on the EPIC array and passed quality control (QC) filtering, and 74/97 were associated with DS at *P* < 0.05 and all with the same direction of effect (Supplementary Data 5).

Finally, an EWAS of *GATA1* mutations (presence/absence) in DS newborns revealed 13 epigenome-wide significant CpGs (Supplementary Data 6), 12 of which were hypomethylated in *GATA1* mutation-positive DS newborns, although all had beta coefficients <0.10. No sex chromosome CpGs were associated with *GATA1* mutations in females or males.

### DMRs associated with DS

We identified 1052 DMRs associated with DS across the genome (Fig. 3b and Supplementary Data 7), following adjustment for variation in cell-type proportions, sex, and ancestry-informative PCs. DMRs were identified on all chromosomes, with a particularly high proportion (11.2%) on Hsa21 (Supplementary Fig. 9). The 1052 DMRs overlapped 943 unique genes, and 291/1052 (27.7%) overlapped promoter regions. The top 20 most significant DMRs (Table 3) overlapped 17 genes, with the top two again including the key hematopoietic TF genes *RUNX1* (*P* = 2.30 × 10^−84^) and *FLI1* (*P* = 1.65 × 10^−78^). The *FLI1* DMR overlapped the promoter of transcript variant 4, which is largely expressed in cord blood megakaryocytes relative to other blood cell types, a pattern not found for other *FLI1* transcript variants in gene expression data in BLUEPRINT (Supplementary Fig. 10)^22^. The top 20 DMRs remained significant following removal of DS newborns with high erythroblasts (*N* = 34) or removal of *GATA1* mutation-positive individuals (*N* = 30), and in ethnicity-stratified analyses with the exception of *CCDC17*, *ANAPC2*, and *MIR1224*, which were not detected in non-Latino whites.

**Table 3:** Top differentially methylated regions (DMRs) associated with Down syndrome.

Chr	Start (hg19)	End (hg19)	Length (bp)	Gene	Mean beta difference^a^	*P* value^b^	Number of CpGs^b^	DMR location	Enhancer overlap^c^	GWAS Catalog SNP overlap (DMR +/−50 Kb)
21	36258497	36259694	1198	*RUNX1*	0.273	2.30 x 10^−84^	11	Promoter	K562, CD34, astrocytes, hippocampus	Eosinophil counts; Eosinophil % of granulocytes; Eosinophil % of white cells; male-pattern baldness; mean corpuscular hemoglobin; red blood cell count; red cell distribution width; sum eosinophil basophil counts
11	128553855	128557589	3735	*FLI1*	0.132	1.65 x 10^−78^	19	Promoter	CD34	Digit length ratio (right hand); Eosinophil counts; height; myopia (pathological); platelet count; plateletcrit; white blood cell count (basophil)
22	51016501	51017723	1223	*CPT1B*	0.210	2.37 x 10^−54^	15	Promoter		Blood metabolite levels; blood protein levels; chronic lymphocytic leukemia; mean corpuscular hemoglobin; mean corpuscular volume; multiple sclerosis; red blood cell count; red cell distribution width; reticulocyte count
7	26577897	26578098	202	*KIAA0087* (lncRNA)	−0.148	5.81 x 10^−49^	2	Gene body		Heart rate response to recovery post exercise; mean corpuscular hemoglobin; red cell distribution width
1	36786285	36788627	2343	*SH3D21*	0.084	2.73 x 10^−47^	10	Enhancer	K562, CD34, astrocytes	Height; lung function (FEV1/FVC)
2	98377791	98378782	992	*TMEM131*	−0.128	1.40 x 10^−43^	5	Enhancer		Autoimmune traits; diastolic blood pressure; educational attainment (years of education); highest math class taken; hypothyroidism; medication use (thyroid preparations)
3	193988507	193988737	231	Intergenic (*HES1* enhancer)	−0.050	8.29 x 10^−41^	3	Enhancer	Hippocampus, brain inferior temporal lobe	None
19	1851750	1851995	246	*KLF16*	0.163	1.42 x 10^−39^	3	Enhancer	CD34, CD19 B cells, hippocampus	Body mass index; cardiovascular disease; cognitive performance; educational attainment (years of education); highest math class taken; lung function (FEV1/FVC); mean corpuscular hemoglobin; mean corpuscular volume; menarche (age at onset); red blood cell count; red cell distribution width
11	124621829	124622348	520	*VSIG2*	−0.095	1.16 x 10^−37^	5	Promoter	CD19 B cells, brain inferior temporal lobe	Autism spectrum disorder or schizophrenia; blood protein levels; cognitive ability, years of educational attainment or schizophrenia (pleiotropy); schizophrenia; smoking initiation
1	46088336	46090107	1772	*CCDC17*	−0.084	5.20 x 10^−34^	11	Promoter		Blood metabolite levels; estimated glomerular filtration rate; height; hemoglobin concentration
2	47382427	47382903	477	*STPG4*	−0.056	7.76 x 10^−34^	7	Promoter		Height
15	40571997	40572794	798	*ANKRD63*	−0.175	3.06 x 10^−32^	5	Intergenic		Height; schizophrenia; type 2 diabetes
15	52944233	52944386	154	*FAM214A*	0.117	5.82 x 10^−30^	4	Promoter	K562, CD34, astrocytes, hippocampus, brain inferior temporal lobe, brain mid frontal lobe	None
3	183959000	183959853	854	*MIR1224*	0.099	7.64 x 10^−30^	9	Promoter		Body mass index; highest math class taken; mean corpuscular hemoglobin; menarche (age at onset)
16	15787920	15787957	38	*NDE1*	−0.105	1.49 x 10^−29^	3	Gene body		Cognitive ability, years of educational attainment or schizophrenia (pleiotropy)
2	54086854	54087552	699	*ASB3*	−0.115	1.98 x 10^−29^	13	Promoter		Anorexia nervosa; heel bone mineral density
9	140066754	140068071	1318	*ANAPC2*	0.012	3.58 x 10^−27^	6	Intergenic		Estimated glomerular filtration rate; height; male-pattern baldness; mean corpuscular hemoglobin; mean corpuscular volume; red blood cell count; reticulocyte count
17	58499679	58500186	508	*C17orf64*	0.059	1.50 x 10^−26^	8	Promoter		None
5	78985434	78986160	727	*CMYA5*	−0.135	8.33 x 10^−26^	10	Promoter		Height
15	75641081	75641171	91	*NEIL1*	0.015	4.19 x 10^−25^	3	Enhancer	CD19 B cells, brain inferior temporal lobe	Estimated glomerular filtration rate; serum uric acid levels
4	81117647	81119473	1827	*PRDM8*	0.174	7.93 x 10^−14^	13	Promoter	Hippocampus, brain inferior temporal lobe	Atrial fibrillation; blood pressure; blood pressure × alcohol consumption interaction; diastolic blood pressure (cigarette smoking interaction); estimated glomerular filtration rate; hypertension; male-pattern baldness

^a^Mean beta difference between DS and non-DS subjects for DMRs calculated by DMRcate.

^b^*P* values (Šidák-corrected) and number of CpGs calculated using the more stringent comb-p method, with the DS EWAS *P* values at each CpG as input.

^c^Limited to enhancers identified in blood or brain tissues^76^.

**Fig. 4:**
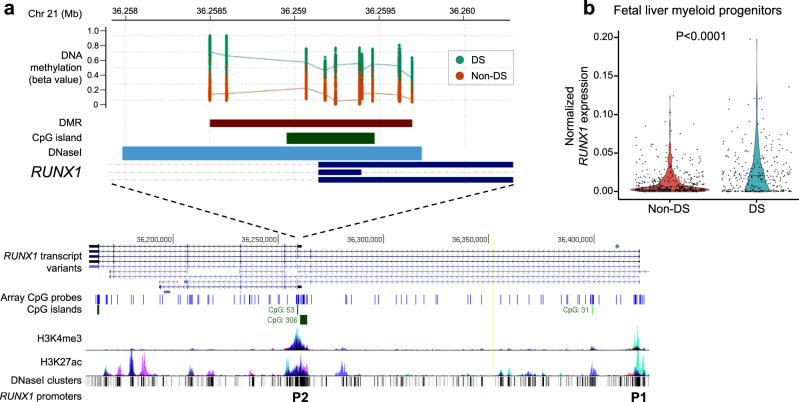
Down syndrome (DS)-associated differentially methylated region (DMR) overlapping *RUNX1* regulatory region. **a** DS-associated DMR (Šidák-corrected *P* value = 2.30 × 10^−84^ from comb-p), which included 11 CpGs, had the greatest mean Δ*β*-value (+0.273) between DS (teal, *N* = 196) and non-DS (red, *N* = 439) newborns, and overlapped a large regulatory region that encompasses the *RUNX1* proximal P2 promoter and the first exon of the P2 isoform, or exon 4 of the P1 isoform. The position of the DMR (brown horizontal bar) is shown relative to the *RUNX1* gene in the UCSC Genome Browser (https://genome.ucsc.edu/), along with tracks for chromatin accessibility (DNase I clusters, darkness corresponds to signal strength) and histone modifications (H3K4me3, H3K27ac), CpG islands (green), and a custom track displaying positions of the array CpG probes (blue). High coverage of CpG probes is shown both at the proximal P2 and distal P1 promoters of *RUNX1*. **b** Violin plot showing normalized *RUNX1* expression derived from single-cell qRT-PCR on index-sorted FL myeloid progenitors with megakaryocyte–erythroid potential (Lin−CD34+CD38+CD45RA−) from non-DS (*N* = 3) and DS (*N* = 3) subjects. *RUNX1* expression was significantly increased in DS myeloid progenitor cells (*N* = 292) compared with non-DS cells (*N* = 412) (*P* = 3.81 × 10^−5^, two-sided Wilcoxon rank-sum test). Horizontal lines represent the median.

**Fig. 5:**
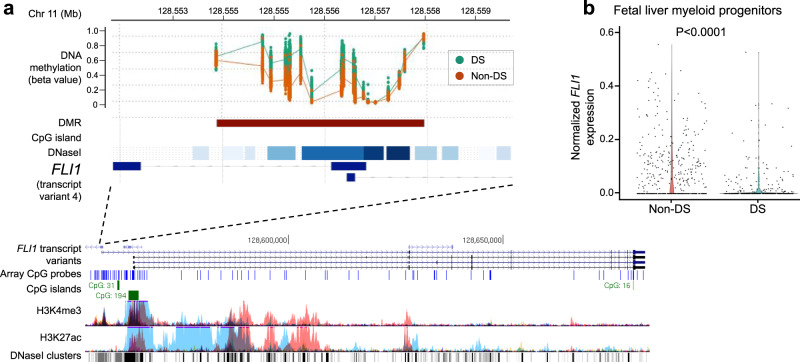
Down syndrome (DS)-associated differentially methylated region (DMR) overlapping *FLI1* promoter region. **a** DS-associated DMR (Šidák-corrected *P* = 1.65 × 10^−78^ from comb-p), which included 19 CpGs, had a mean Δ*β*-value = +0.132 between DS (teal, *N* = 196) and non-DS (red, *N* = 439) newborns, and overlapped a promoter of *FLI1* transcript variant 4. The position of the DMR (brown horizontal bar) is shown relative to the *FLI1* gene in the UCSC Genome Browser (https://genome.ucsc.edu/), along with tracks for chromatin accessibility (DNase I clusters, darkness corresponds to signal strength) and histone modifications (H3K4me3, H3K27ac), CpG islands (green), and a custom track displaying positions of the array CpG probes (blue). **b** Violin plot showing normalized *FLI1* expression derived from single-cell qRT-PCR on index-sorted FL myeloid progenitors with megakaryocyte–erythroid potential (Lin−CD34+CD38+CD45RA−) from non-DS (*N* = 3) and DS (*N* = 3) subjects. *FLI1* expression was significantly reduced in DS myeloid progenitor cells (*N* = 292) compared with non-DS cells (*N* = 412) (*P* = 2.20 × 10^−16^, two-sided Wilcoxon rank-sum test). Horizontal lines represent the median.

Of six DMRs with at least ten CpG probes and with mean Δ*β*-value >0.10 (Table 3), two overlapped regulatory regions in *RUNX1* (Fig. 4a) and *FLI1* (Fig. 5a), and the remaining four DMRs overlapped promoter regions of genes involved in brain development (*CPT1B*, *CMYA5*, and *PRDM8*) and the immune system (*ASB3*) (Supplementary Fig. 11 and Table 3). Assessment of genome-wide association study (GWAS) Catalog SNPs revealed that 8 of the top 20 DMRs are nearby SNPs associated with hematological traits, with 7 DMRs nearby SNPs associated with brain-related traits (Table 3 and Supplementary Data 8).

We next assessed overlap between our DS-associated DMRs with those reported in the previous largest EWAS of DS^16^. Of the 66 DMRs previously identified with a large *β*-value difference (>0.15), we also detected 37 as DMRs, all of which had concordant Δ*β*-value directions (Supplementary Data 5).

In addition, we identified 59 DMRs associated with *GATA1* mutations in DS neonates (Supplementary Data 6); the most significant region encompassed the noncoding RNA *VTRNA2-1* (*P* = 1.71 × 10^−20^), with reduced DNA methylation (mean Δ*β*-value = −0.115) in *GATA1* mutation-positive DS newborns versus wild-type DS newborns. DNA methylation in DS newborns at the top two DS-associated DMRs, in *RUNX1* and *FLI1*, was not driven by the presence of *GATA1* mutations; in fact, at both DMRs, which were significantly hypermethylated in DS newborns, the mean methylation levels were slightly lower in *GATA1* mutation-positive than in wild-type DS newborns, albeit still considerably higher than in non-DS newborns (Supplementary Figs. 12 and 13).

### Gene expression changes in DS versus non-DS FL CD34+ cells

**Fig. 6:**
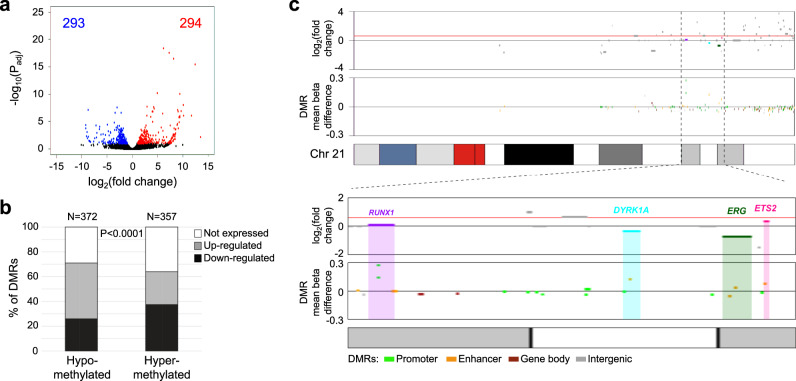
Correlation between differential methylation in newborn dried bloodspots and differential gene expression in fetal liver CD34+ cells in Down syndrome (DS) and non-DS samples. **a** Volcano plot showing differential gene expression between DS (*N* = 3) and non-DS (*N* = 3) FL CD34+ cells, with significantly upregulated and downregulated genes (FDR-adjusted *P* < 0.1) highlighted in red (*N* = 294) and blue (*N* = 293), respectively. **b** Bar chart showing the number of hypomethylated (*N* = 372) and hypermethylated (*N* = 357) DS-associated differentially methylated regions (DMRs) that overlapped gene promoters/enhancers, and the direction of differential expression of the corresponding genes in DS versus non-DS FL CD34+ cells. For hypomethylated DMRs, 96 genes were downregulated and 167 genes upregulated (109 genes not expressed), whereas for hypermethylated DMRs, 134 genes were downregulated and 94 genes upregulated (129 not expressed), a difference that was highly significant in a two-sided Fisher’s exact test (*P* = 9.20 × 10^−7^). **c** DS-associated DMR methylation levels and corresponding differential gene expression levels across chromosome 21. The zoomed-in plot below highlights four genes that play an important role in hematopoiesis, *RUNX1*, *DYRK1A*, *ERG*, and *ETS2*, all of which were overlapped by hypermethylated DMRs in promoters/enhancers and also demonstrated less than the expected 1.5-fold change in expression (indicated by the red horizontal line) in DS compared to normal FL CD34+ cells.

FL is the main site of hematopoiesis until birth and neonatal blood is likely derived from FL HSPC. To ascertain whether differences in genome-wide DNA methylation found in neonatal T21 blood cells correlate with differences in gene expression, we analyzed RNA-sequencing data from DS (*N* = 3) and non-DS (*N* = 3) FL HSPC. We found 587 significantly differentially expressed genes between DS and non-DS FL CD34+ cells (FDR < 0.1), of which 294 genes were upregulated and 293 downregulated in DS (Fig. 6a and Supplementary Data 9). DS-associated DMRs identified at promoter or enhancer regions in neonatal blood (*N* = 729, Supplementary Data 7) overlapped 491 genes that demonstrated any change in expression in DS FL cells compared with non-DS cells; hypermethylation at these DMRs correlated with decreased gene expression in DS FL CD34+ cells, whereas hypomethylation correlated with increased gene expression (*P* < 0.0001, two-tailed Fisher’s exact test) (Fig. 6b and Supplementary Data 7), a relationship that remained when limiting to the significantly differentially expressed genes (*P* = 0.0002) or after excluding Hsa21 genes (*P* < 0.0001). Conversely, no relationship between hyper-/hypomethylation and gene expression was found for the 323 DMRs outside of promoters/enhancers (Supplementary Fig. 14).

We next took a closer look at DMRs and gene expression on Hsa21. While an extra copy of Hsa21 would predict for a 1.5-fold increased expression of the genes on this chromosome, this is often not the case suggesting that epigenetic mechanisms regulate gene expression in the context of aneuploidy. Here, as for non-Hsa21 genes, we found that Hsa21 changes in gene expression in DS fetal hematopoietic cells negatively correlated with DNA methylation status at promoter/enhancer regions in DS neonatal hematopoietic cells (Fig. 6c and Supplementary Fig. 14). Several Hsa21 genes that play a key role in hematopoiesis, such as *RUNX1*, *ERG, DYRK1A*, and *ETS2* showed less than the expected 1.5-fold change when compared to normal FL, and this was accompanied by significant hypermethylation at promoters/enhancers of these genes in neonatal blood (Supplementary Data 7 and Fig. 6c).

Finally, we analyzed the expression of *RUNX1* and *FLI1*, the top 2 genes with DMRs and both essential regulators of megakaryopoiesis, via single-cell qRT-PCR on index-sorted DS and non-DS FL myeloid progenitors with megakaryocyte–erythroid potential (Lin−CD34+CD38+CD45RA−). This showed markedly lower expression of *FLI1* in DS FL myeloid progenitors (*P* < 0.0001), consistent with hypermethylation of the *FLI1* promoter in DS neonatal blood (Fig. 5b), while *RUNX1* expression was increased in DS myeloid progenitors (*P* < 0.0001) compared to normal FL counterparts (Fig. 4b), perhaps reflecting the significant DMR hypermethylation found at the *RUNX1* P2 promoter but not the P1 promoter in DS newborns (Supplementary Fig. 7).

## Discussion

We report the results from the largest, and first multiethnic, EWAS of DS in blood cell samples at birth, confirming several known loci and identifying many novel regions, including at *FLI1*, that were significantly differentially methylated in newborns with DS compared with newborns without DS. The 652 epigenome-wide significant CpGs and 1052 DMRs demonstrate the profound epigenome-wide consequences of T21, which likely contribute toward phenotypic variation in DS. The majority of DS-associated DNA methylation changes were found on euploid (non-21) chromosomes, as previously reported^15–17^, supporting that T21 results in genome-wide perturbations in gene regulation^12,23^. Indeed, our results from RNA sequencing of fetal DS and non-DS HSPCs support the early-life, genome-wide perturbation of gene expression in hematopoietic cells that broadly correlates with differential DNA methylation patterns in DS.

The effects of T21 on the function of *RUNX1*, a crucial regulator of hematopoiesis particularly in early development^24^, appear complex. Although DS was largely associated with hypomethylation on Hsa21, we found significant hypermethylation at *RUNX1*, as reported previously in DS and in DS-ALL^16–18,25^. We note that *RUNX1* hypermethylation in DS was specific to the proximal P2 promoter^26^, which is thought to be the dominant regulator of *RUNX1* expression during embryonic development, driving formation of the hemogenic endothelium and early hematopoiesis^27,28^. Dosage of *RUNX1* during these early stages is tightly controlled^29,30^, suggesting that RUNX1 downregulation via P2 promoter hypermethylation may be required for viable embryo development in DS. The distal P1 promoter becomes active once cells commit to the hematopoietic lineage and is the predominant promoter in definitive hematopoiesis^27,28^, consistent with the pattern of *RUNX1* expression we observed in DS FL myeloid progenitors. Promoter switching from P2 to P1 involves changes in DNA methylation at the P1 promoter but not at P2, which was found to be unmethylated across cell types^31^, suggesting that P2 hypermethylation is unique to DS.

The most significant DMR outside of Hsa21 overlapped *FLI1*, another important regulator of megakaryopoiesis^32,33^, specifically at the promoter of transcript variant 4 that is mainly expressed in megakaryocytes. FLI1 protein is a critical binding partner of both RUNX1 and GATA1 during terminal megakaryocyte maturation^34,35^ and all three proteins cooperate in transcriptional control of megakaryocyte differentiation^36^. Similar to *RUNX1*, germline loss of *FLI1* has been associated with thrombocytopenia^33^, defects in megakaryopoiesis^37^, and familial platelet disorders^38^. We report that *FLI1* expression is significantly reduced in DS FL myeloid progenitor cells. Our results support that T21 leads to epigenetic dysregulation of both *RUNX1* and *FLI1*, which may contribute toward abnormal megakaryocyte development in DS FL cells^3^, and to the development of TAM and the concomitant risk of AMKL in DS infants. The etiology and timing of these epigenetic changes remain to be determined. Along with *RUNX1*, *FLI1* is also a critical regulator of embryonic hematopoiesis^24,39^; thus, compensatory epigenetic downregulation of *RUNX1* and *FLI1* may be required for viable embryogenic development in DS, but potentially also results in increased risk of hematological malignancies.

Our results confirm previous studies that pinpointed *RUNX1* as one of the most differentially methylated genes in blood in individuals with DS^16,17^. It is interesting that DS-associated hypermethylation at *RUNX1* has also been reported in DS brain tissue^18,40^, supporting the early fetal origins of these epigenetic changes and potential pleiotropic effects on DS phenotypes. Indeed, *RUNX1* has been shown to play a role in proliferation and differentiation of select neural progenitor cells, including in hippocampal precursor cells^41,42^. The overlap of DS-associated DMRs with GWAS loci for both cognitive-related and hematological traits, such as at *KLF16*, further supports the possibility that epigenetic dysregulation may underlie both hematologic defects and cognitive development in DS. Remarkably, two of the most significant DMRs, overlapping promoters of *CPT1B* and *CMYA5*, were recently associated with hippocampal volume in non-DS individuals; for both DMRs, the direction of DNA methylation changes in DS newborns was associated with smaller hippocampal volume^43^. Additional DS-associated DMRs overlapped *NDE1*, *PRDM8*, and the enhancer locus for *HES1*, genes that all play an important role in neurogenesis^44–46^.

Cell-type deconvolution revealed that DS newborns had relatively high proportions of erythroblasts, possibly indicative of intrauterine or perinatal hypoxia^47^, pulmonary hypertension^48^, or TAM^49^. Although no DS newborns in this study developed childhood leukemia^50^, targeted *GATA1* sequencing identified that ~14% harbored a likely functional somatic *GATA1* mutation, consistent with the observation that the majority of DS newborns with TAM and Silent TAM will not develop AMKL^8^. We found significant association between *GATA1* mutations and higher erythroblast proportions; however, almost two-thirds of DS newborns with high erythroblast proportions did not harbor *GATA1* mutations, suggesting a greater role for pre- and perinatal hypoxic conditions in contributing to this phenotype.

Nonfunctional *GATA1* variants tended to have much lower VAF than functional ones, supporting that *GATA1*-truncating mutations confer a growth advantage to fetal hematopoietic cells and are clonally selected during development of TAM. Moreover, the true frequency at which somatic *GATA1* mutations arise in utero may be higher than detected at the current limits of detection and by sampling blood at birth. The etiology of *GATA1* mutations in DS remains unknown, but is potentially related to T21-associated upregulation of GATA1^3^; increased transcription is a known cause of DNA mutagenesis^51^. Intriguingly, human adaptation to hypoxic conditions includes upregulation of GATA1 to drive erythropoiesis^52^. Thus, hypoxic intrauterine conditions in developing DS fetuses may contribute to the generation of *GATA1* mutations or at least to expansion of mutant *GATA1* clones^53^. Our EWAS of *GATA1* mutations in DS revealed a DMR overlapping *VTRNA2-1*, a metastable epiallele at which DNA methylation levels were previously associated with the periconceptional environment^54^, suggesting a potential environmental role in the development of *GATA1* mutations.

An important strength of our study was the use of newborn-dried bloodspots (DBS), which increased our power to detect differentially methylated loci associated with DS, as epigenetic influences of environmental exposures and age-related changes as well as drift would be much reduced compared with studies in older individuals. Our study does have some limitations. Although DBS biospecimens were all obtained from newborns in California, we did not match DS and non-DS newborns by demographic variables such as sex, race/ethnicity, or birth year. This should not have biased our findings, however, as our EWAS was adjusted for sex and principal components, and similar results were found in Latinos and non-Latino whites. Second, analytical tools such as ReFACTor and cell-type deconvolution were developed in euploid individuals, although we did confirm some known differences in blood cell proportions (using conventional cell enumeration methods) between DS and non-DS individuals. Reference-free adjustment for cell-type composition was performed in our EWAS, given the highly significant differences in estimated blood cell proportions and to maximize our power to detect epigenetic changes associated with trisomy 21; however, we cannot rule out that some of the DNA methylation changes associated with DS may reflect differences in peripheral blood cell composition between DS and non-DS newborns, and future studies should explore the epigenetic effects of DS in sorted blood cells. Finally, our study was limited to newborn whole-blood samples, and would not detect tissue-specific DNA methylation differences outside of blood that may underlie DS-related phenotypes. Studies have, however, demonstrated similarities in the epigenetic effects of T21 across tissues^40^, and in the use of blood DNA methylation as a biomarker for traits in other tissues, such as brain-related phenotypes^43^.

Our results demonstrate the profound genome-wide effects of T21 on DNA methylation, with important implications for the defects in hematopoiesis, cognition, immune function, and other developmental processes that arise in individuals with DS. Determining the etiologies of these epigenetic changes will be essential to understand and potentially ameliorate DS phenotypes. Epigenetic changes in DS may occur due to triplication of specific genes on Hsa21, such as *HMGN1*^23^ or *DNMT3L*^55^, the effects of additional genomic material on three-dimensional chromatin organization, or via some compensatory mechanism triggered early in DS fetal development. One might predict compensatory hypermethylation of triplicated genes; thus, it is also important to understand why Hsa21 is largely hypomethylated in DS, and how this hypomethylation is distributed across the three copies of Hsa21. Finally, case–control studies within DS populations are required to investigate the association between epigenetic variation across tissues and the variable penetrance and expressivity of DS-related phenotypes.

## Methods

### Study subjects

This study was approved by Institutional Review Boards at the California Health and Human Services Agency, University of Southern California, and University of California Berkeley, and by Hammersmith and Queen Charlotte’s Hospital Research Ethics Committee (ref 04/Q0406/145). The deidentified newborn DBS from the California Biobank Program for this project (SIS request numbers 572 and 600) were obtained with a waiver of consent from the Committee for the Protection of Human Subjects of the State of California. FL samples were obtained with written consent.

DBS were obtained from 198 DS newborns, without a leukemia diagnosis by 15 years of age, from the California Biobank Program via linkage between the California Department of Public Health Genetic Disease Screening Program and California Cancer Registry^50^. We also obtained newborn DBS from 442 non-DS (cancer-free) children from the California Biobank Program^56^. Demographic and birth-related data for subjects that passed QC are summarized in Table 1. The majority of individuals were reported as Latino (*N* = 357) or non-Latino white (*N* = 178), and the remainder as African American (*N* = 34), Asian/Pacific Islander (*N* = 55), or other (*N* = 11).

A separate sample set was used for gene expression studies. Second-trimester FL samples were collected during elective surgical termination of pregnancy and processed immediately. Donated fetal tissue was also provided by the Human Developmental Biology Resource (www.hdbr.org) regulated by the UK Human Tissue Authority (www.hta.gov.uk).

### Genome-wide DNA methylation arrays

DNA was extracted from one-third portions of each newborn DBS using the Qiagen DNA Investigator blood card protocol, and bisulfite conversion performed using Zymo EZ DNA Methylation kits. Bisulfite-converted DNA samples from DS and non-DS newborns were block-randomized (ensuring equivalent distribution of sex and race/ethnicity on all plates) and run on Illumina Infinium MethylationEPIC Beadchip genome-wide DNA methylation arrays.

### DNA methylation array data processing, visualization, and annotation

For QC assessment of DNA methylation array data, we imported raw IDAT files into R and used the “minfi” package to calculate mean detection *P* values using the “detectionP” function. Further data QC and normalization were performed using the R package “SeSAMe,” with background correction using “noob” and using *P* value with out-of-band (OOB) array hybridization for removal of poor-performing probes, accounting for deleted and hyperpolymorphic regions (R version: 3.6.0)^57,58^. The R package “conumee” was used to generate copy-number variation (CNV) plots for all subjects to check T21 status^59^, with twenty randomly selected non-DS newborns used to construct a CNV reference, and subjects were removed if the reported DS status did not match T21 status based on visual inspection (one “control” appeared to have T21 and was excluded). Subjects with missingness >5% (2 DS and 2 non-DS) were removed, resulting in a final study number of 196 DS and 439 non-DS newborns. CpG probes with missingness >5% were subsequently removed (*N* = 137,060), and the remaining missing values imputed using “impute.knn” function from the “impute” package.

We removed probes located on chromosomes X and Y as well as CpGs located at SNP sites with a minor allelic frequency >5%, resulting in a final CpG probe set *N* = 651,772. Using data from this final set of probes, and also excluding probes on Hsa21, we calculated PCs in R using the “prcomp” command to create PCA plots. Additional dimensional reduction plots to visualize clustering within samples were generated using the t-SNE algorithm, with the “Rtsne” package^60^. Betas were converted to *M* values for construction of heatmaps using the “ComplexHeatmap” package^61^ for the 2000 CpGs with the greatest mean absolute deviation across chromosomes, excluding 21, X, and Y, and annotated for DS status, sex, and deconvoluted blood cell proportions.

All CpGs in the EWAS results were annotated using the annotation database from the “IlluminaHumanMethylationEPICanno.ilm10b4.hg19” package in R^62^. DMRs of interest were visualized using the “coMET” package^63^.

### Assessment and adjustment of cell-type heterogeneity

Reference-based deconvolution of blood cell proportions in DS and non-DS newborns was performed using the Identifying Optimal Libraries algorithm^64,65^. We used the “estimateCellCounts2” function in the R package “FlowSorted.Blood.EPIC” and DNA methylation data from cord blood cell reference samples in the R package “FlowSorted.CordBloodCombined.450k,” to estimate proportions of monocytes, granulocytes, natural killer cells, B lymphocytes, T lymphocytes (both CD4^+^ and CD8^+^), and nRBC/erythroblasts in newborns^66,67^. We performed separate linear regression tests with each blood cell-type proportion as the dependent variable and DS status as the independent variable, adjusting for plate, sex, the first ten EPISTRUCTURE PCs to account for genetic ancestry (see below), gestational age, age at DBS collection, and birth weight. We also tested the association of each blood cell-type proportion with birth-related and demographic variables. Within DS newborns, we performed additional regression models to test the association between *GATA1* mutations, treated either as a binary variable (presence/absence) or a linear variable (i.e., VAF), and blood cell proportions and birth variables, adjusting for plate, sex, and EPISTRUCTURE PCs as above.

To account for cell-type heterogeneity in the EWAS, we obtained Reference-Free Adjustment for Cell-Type composition (ReFACTor) PCs using the GLINT tool (v1.0.4)^68,69^, with the assumed number of cell types in the data (**k**) set to 7 to align with the reference-based approach described above, and with adjustment for plate and sex.

### *GATA1* mutation sequencing in DS newborns

We performed targeted sequencing of *GATA1* in 184 DS newborns with sufficiently remaining DNA isolated from DBS, using methods modified from previously described protocols^8,70^. In brief, targeted amplification of *GATA1* from isolated genomic DNA was performed in tandem, with the addition of sample barcodes and sequencing adapters. Six primer pairs generating 150–210-bp amplicons covering the entirety of exon 2 and the first 115 bases of exon 3 and including three exon/intron boundaries were individually amplified in quadruplicate following Fluidigm’s Access Array^TM^ IFC 4-Primer Amplicon Tagging Workflow (Fluidigm: PN 68000161). Primer sequences are included in Supplementary Table 4. After amplification, 2 μl for each sample were pooled and PCR products purified using AMPureXP beads (Beckman Coulter). Quality and size distribution were determined using a Tapestation system (Agilent Technologies). Library concentration was determined by the Qubit dsDNA HS Assay kit (Thermo Fisher). Sequencing was performed on an Illumina MiSeq as 150 base-paired-end reads. Mapping and variant analysis were performed using an in-house pipeline generating VarScan somatic data^71^, as previously described^8,70^. VAF were manually verified and compared to in-run controls using the Integrative Genomics Viewer for visualization^72^. The limit of detection of mutant *GATA1* sequence was 0.3–2% depending on read quality and depth of sequencing.

### Epigenome-wide association analyses

GLINT was used to obtain EPISTRUCTURE PCs, adjusting for the first ten ReFACTor PCs, to account for genetic ancestry^73^. CpG probes on chromosomes X and Y, and at SNP sites with MAF > 5% were removed, resulting in a final probe set *N* = 651,772. A multiethnic EWAS of DS was performed using linear regression in R, with each CpG *β*-value as the dependent variable and DS status the independent variable, adjusting for sex, plate, the first ten ReFACTor PCs to adjust for cell-type proportions, and first ten EPISTRUCTURE PCs to adjust for genetic ancestry.

For sensitivity analysis to account for potential confounding of uncorrected population stratification in the overall multiethnic EWAS, we repeated the EWAS separately in the two largest self-reported race/ethnicity groups, in Latinos (DS *N* = 104, non-DS *N* = 256) and non-Latino whites (DS *N* = 54, non-DS *N* = 124). In these race/ethnicity-stratified EWAS models, we only adjusted for the first six EPISTRUCTURE PCs to reach acceptable levels of epigenomic inflation (*λ* = 1.46 for Latinos and 1.09 for non-Latino whites). In addition, we repeated the multiethnic EWAS following: (1) removal of the separate cluster of DS individuals (*N* = 34) observed in our visualization plots and with high nRBC proportions in the cell-type deconvolution analyses (Figs. 1 and 2), or (2) removal of DS newborns with any somatic *GATA1* mutations identified by sequencing (*N* = 30).

To explore epigenetic changes associated with *GATA1* mutations, we performed a separate EWAS within DS newborns with *GATA1* mutation status as a binary dependent variable. Bonferroni correction was applied to correct for multiple testing (*P* < 7.67 × 10^−8^, based on 651,772 CpGs).

Gene pathway enrichment analyses were performed for genes overlapped by epigenome-wide significant CpGs using the “methylglm” function in the R package “methylGSA,” with assessment of GO and KEGG pathways^74^. We used a gene-list minimum size of 10 and maximum size of 500, and only considered pathways with an FDR-corrected *P* value < 0.05 as significant.

We also performed enrichment analysis to assess the significant overlap between epigenome-wide significant CpGs and genomic locations (i.e., promoters, exons, introns, 1–5 Kb, 3′-UTRs, 5′-UTRs, intergenic, intron/exon boundaries, CpG islands, shelves, shores, and open sea), as well as functional features, including TF-binding sites, histone modification markers, DHS, and predicted enhancer regions. The number of significant and nonsignificant CpGs overlapping each feature was compared by the Fisher’s exact test. For TF-binding sites, we included all available TFs (*N* = 161) in the ENCODE ChiP-seq database for the K562 cell line (wgEncodeRegTfbsClusteredV3.bed). Histone modification data were downloaded for primary HSCs (cell line E035) from the Roadmap Epigenomics Mapping Consortium database^75^. DHS sites were downloaded from the ENCODE project (wgEncodeRegDnaseClusteredV3.bed file). Finally, we assessed overlap with previously identified enhancer regions for three HSC cell lines (BI_CD34_Primary_RO01536, BI_CD34_Primary_RO01480, and BI_CD34_Primary_RO01549), CD19^+^ B cells (CD19_primary), GM12878 lymphoblastoid cells, K562 cells, and four brain cell lines (astrocytes, frontal lobe cells, temporal lobe cells, and hippocampus cells)^76^. All enrichment analyses were performed separately for hyper- and hypomethylated CpGs, and Bonferroni correction for multiple testing was applied as warranted for each analysis based on the number of comparisons.

DMRs associated with DS were identified using two different methods, DMRcate and comb-p^77,78^. The *P* values obtained in each EWAS were used for comb-p. DMRcate was run with adjustment for cell-type heterogeneity using the first ten ReFACTor PCs, as well as for sex, plate, and the first ten EPISTRUCTURE PCs (except in race/ethnicity-stratified analyses below). We retained DMRs that spanned a minimum of two CpGs, had a maximum distance of 1000 bp between methylation peaks, had an FDR-corrected *P* value < 0.01 in DMRcate, had a Šidák-corrected *P* value < 0.01 in comb-p, and that displayed any overlap between the coordinates of regions called by DMRcate and comb-p.

In the analyses of DMRs in DS, we generated DMR calls for (i) overall DS versus non-DS subjects, (ii) Latino and (iii) non-Latino white-stratified analyses (in both of which the first six EPISTRUCTURE PCs were used to adjust for genetic ancestry, as for the EWAS models above), (iv) following removal of the 34 DS newborns with high nRBC proportions, (v) following removal of the 30 DS newborns with *GATA1* mutations, and (vi) for *GATA1* mutation status as described for the EWAS of *GATA1* mutations. To assess the potential functions of genes overlapped by the most significant DMRs in the overall analysis of DS, we investigated the presence of SNPs, and their corresponding trait associations, in the NHGRI-EBI Catalog of published GWAS and with reported *P* values < 5.0 × 10^−8^ in regions spanning +50 and −50 Kb of each DMR locus^79^.

### Gene expression analysis in DS and non-DS FL CD34+ cells by bulk RNA sequencing

*GATA1* mutation analysis, CD34+ separation, and immunohistochemistry were performed as previously described^3,80^. Fluorescence in situ hybridization was used to confirm the presence (*N* = 3) or absence (*N* = 3) of T21 in FL. Bulk RNA sequencing of DS and non-DS FL cells was performed using the SMART-Seq2 protocol^81^. In brief, 100 purified HSC or progenitor cells (HSPCs) from gestation-matched DS (*N* = 3) and non-DS (*N* = 3) 2nd-trimester FL samples were sorted directly into lysis buffer containing 0.4% Triton X-100 (Sigma-Aldrich), RNase inhibitor (Clontech), 2.5-mM dNTPs (Thermo Fisher), and 2.5-μM oligo-dT30VN primer (Biomers.net). cDNA was generated using SuperScript II (Invitrogen), preamplified using KAPA HiFi HotStart ReadyMix (KAPA Biosystems) using 18 cycles of amplification. After PCR amplification, the cDNA libraries were purified with AMPure XP beads (Beckman Coulter) according to the manufacturer’s instructions. Post-purification libraries were resuspended in EB buffer (Qiagen). The quality of cDNA traces was assessed by using a High Sensitivity DNA Kit in a Bioanalyzer instrument (Agilent Technologies). Library preparation was performed using the Nextera XT DNA Library Preparation Kit (Illumina) according to the manufacturer’s instructions. Indexed cDNA libraries were multiplexed and sequenced using Illumina HiSeq2500 to generate 150-bp paired-end reads, yielding >30 million reads per sample.

Following sequencing, QC analysis was conducted using the fastQC package (http://www.bioinformatics.babraham.ac.uk/projects/fastqc). Reads were mapped to the human genome assembly hg19 using STAR software^82^. Quality and adapter trimming were performed using TrimGalore (https://github.com/FelixKrueger/TrimGalore). The featureCounts function from the Subread package in R was used to quantify gene expression levels using standard parameters. We excluded from our analyses any genes with <100 combined reads across all six samples. Differential gene expression between groups was assessed using the DESeq2 package^83^.

### Single-cell gene expression analysis in DS and non-DS FL CD34+ cells

Single-cell analysis of *RUNX1* and *FLI1* gene expression was performed by reverse transcription (RT)-qPCR using the Biomark HD microfluidics system (Fluidigm). Flow cytometry was performed as previously described^84^. Samples were FACS-sorted using BD Fusion instruments, and data analyzed using FlowJo software. Single cells from FL common myeloid progenitors (CMP:Lin−CD34+CD38+CD45RA−CD123+) and megakaryocyte–erythroid progenitors (MEP:Lin−CD34+CD38+CD45RA−CD123−) from three DS and three non-DS subjects were index-sorted into a 96-well plate containing 5 μL of preamplification mix, which contained One-Step RT-PCR System with Platinum Taq kit (Invitrogen), SUPERASE-In RNase inhibitor (Ambion), low EDTA TE buffer (Invitrogen), and 0.2X Taqman assay mastermix. Plates were sealed, briefly centrifuged, and cDNA synthesis and sequence-specific preamplification performed (three-step PCR of step 1, reverse transcriptase at 50 °C for 15 min, step 2, inactivation of RTase, activation of Taq at 95 °C for 2 min, and step 3, specific target amplification at 95 °C for 15 s, then 60 °C for 4 min repeated for 20 cycles). Preamplified products were diluted by adding 20 μL of low EDTA TE buffer, and samples then analyzed using Universal PCR Master Mix (Applied Biosystems) and individual Taqman gene expression assays (RUNX1 Assay ID: Hs01021970_m1, FLI1 Assay ID: Hs00956711_m1 [Life Technologies]), on the Biomark System (Fluidigm) using the 96.96 Dynamic Arrays as per the manufacturer’s protocol. Sorted cells were simultaneously analyzed for relative expression levels of *RUNX1* and *FLI1*. Gene expression was normalized to the average expression of three housekeeping genes *B2M* (Assay ID: Hs00984230_m1)*, GAPDH* (Hs02758991_g1), and *ACTB* (Assay ID Hs01060665_g1)^84^.

### Reporting summary

Further information on research design is available in the Nature Research Reporting Summary linked to this article.

## Supplementary information

Supplementary Information

Peer Review File

Reporting Summary

Description of Additional Supplementary Files

Supplementary Data 1

Supplementary Data 2

Supplementary Data 3

Supplementary Data 4

Supplementary Data 5

Supplementary Data 6

Supplementary Data 7

Supplementary Data 8

Supplementary Data 9

## Data Availability

ENCODE TF-binding site dataset wgEncodeRegTfbsClusteredV3.bed.gz was downloaded from the UCSC Genome Browser (https://hgdownload.cse.ucsc.edu/goldenpath/hg19/encodeDCC/wgEncodeRegTfbsClustered/). Histone modification data were downloaded for primary HSCs (cell line E035, CD34 primary cells) from the Roadmap Epigenomics Mapping Consortium database (https://egg2.wustl.edu/roadmap/data/byFileType/peaks/). ENCODE DNase I hypersensitive site data are available at http://hgdownload.cse.ucsc.edu/goldenpath/hg19/encodeDCC/wgEncodeRegDnaseClustered/. NHGRI-EBI GWAS Catalog data are available at https://www.ebi.ac.uk/gwas/docs/file-downloads. FLI1 transcript variant data were downloaded from the BLUEPRINT Consortium Blood Atlas (https://blueprint.haem.cam.ac.uk/mRNA/). This study used biospecimens from the California Biobank Program. Any uploading of genomic data (including genome-wide DNA methylation data) and/or sharing of these biospecimens or individual data derived from these biospecimens has been determined to violate the statutory scheme of the California Health and Safety Code Sections 124980(j), 124991(b), (g), (h), and 103850 (a) and (d), which protect the confidential nature of biospecimens and individual data derived from biospecimens. The individual-level data derived from these biospecimens and that support the findings of this study are available from the corresponding author upon request, and with permission from the California Biobank Program. RNA-seq data from DS and non-DS FL CD34+ cells have been deposited at the Gene Expression Omnibus (GEO) with accession code: GSE160637. Source data are provided with this paper.

## Source data

Source Data
