# Supplementary material for: The genome-wide impact of trisomy 21 on DNA methylation and its implications for hematopoiesis
Source: Nat Commun. 2021 Feb 5;12:821. doi: 10.1038/s41467-021-21064-z (PMC7865055; doi:10.1038/s41467-021-21064-z)
Supplement: Supplementary file 1 — Supplementary Information [file 41467_2021_21064_MOESM1_ESM.pdf]

The genome-wide impact of trisomy 21 on DNA methylation and its implications for hematopoiesis

SUPPLEMENTARY MATERIAL

This file includes:

- Supplementary Tables 1-4
- Supplementary Figures 1-14

Supplementary Table 1 – Baseline characteristics of newborns with Down syndrome, with and without somatic *GATA1* mutations.

	GATA1 mutation +ve (N=30) N (%)	*GATA1* Wildtype (N=154) N (%)	P (binary)*	P (continuous)**
**Sex**				
Female	17 (56.7%)	84 (54.5%)	0.38	0.46
Male	13 (43.3%)	70 (45.5%)		
**Race/ethnicity**				
Latino	20 (66.7%)	76 (49.4%)	0.52	0.38
Non-Latino white	7 (23.3%)	43 (27.9%)		
Non-Latino black	2 (6.7%)	19 (12.3%)		
Asian	1 (3.3%)	16 (10.4%)		
**Blood collection age (days)**				
Mean (SD)	2.53 (±1.61)	2.45 (±2.13)	0.61	0.63
Median (range)	2.25 (0.21-6.04)	1.67 (0.17-15.25)		
Missing	1 (3.3%)	2 (1.3%)		
**Gestational age (weeks)**				
Mean (SD)	37.8 (±2.2)	38.3 (±2.2)	0.43	0.19
Median (range)	38.1 (32.6-41.1)	38.3 (26.4-44.7)		
Preterm (<37 weeks, N)	10 (37.0%)	26 (16.9%)	0.033 ***	
Missing	3 (10.0%)	13 (8.4%)		
**Birth weight (kg)**				
Mean (SD)	2.90 (±0.56)	3.06 (±0.75)	0.71	0.57
Median (range)	2.93 (1.61-3.98)	3.05 (0.96-8.65)		
Small-for-gestational age	6 (23.1%)	23 (16.9%)	0.42 ***	
Missing	1 (3.3%)	3 (1.9%)		

* P-values calculated using logistic regression, including *GATA1* mutation as a binary variable (presence/absence).

** P-values calculated using linear regression, including *GATA1* mutation variant allele fraction (VAF) as a continuous variable.

*** P-value calculated using two-sided Fisher's exact test.

P-values were not adjusted for multiple comparisons.

Abbreviations: kg: kilogram, SD: standard deviation.

Supplementary Table 2 – Deconvoluted blood cell proportions by Down syndrome status and *GATA1* mutation status.

Deconvoluted cell type	Down syndrome (DS) status				*GATA1* mutation status					
	DS (N=196) Mean (SD); Median (range)	Non-DS (N=439) Mean (SD); Median (range)	Coefficient (SE)	*P*	Mutation +ve (N=30) Mean (SD); Median (range)	Wildtype (N=154) Mean (SD); Median (range)	Coefficient (SE), binary*	*P* (binary)*	Coefficient (SE), continuous**	*P* (continuous)**
$CD8^+$ T-cells	0.053 (0.032); 0.047 (0-0.190)	0.031 (0.022); 0.028 (0-0.168)	0.006 (0.003)	0.073	0.043 (0.035); 0.041 (0-0.124)	0.053 (0.030); 0.047 (0-0.190)	-0.006 (0.005)	0.24	-0.047 (0.013)	4.57E-04
$CD4^+$ T-cells	0.119 (0.074); 0.106 (0-0.431)	0.172 (0.058); 0.165 (0.031-0.428)	-0.087 (0.005)	2.26E-53	0.152 (0.092); 0.105 (0-0.431)	0.110 (0.068); 0.095 (0-0.387)	0.041 (0.009)	2.17E-05	0.144 (0.024)	2.30E-08
B-cells	0.008 (0.011); 0.003 (0-0.051)	0.040 (0.021); 0.038 (0-0.119)	-0.031 (0.003)	3.48E-28	0.005 (0.008); 0.003 (0-0.051)	0.009 (0.012); 0.004 (0-0.051)	-0.004 (0.002)	0.13	-0.016 (0.007)	0.014
NK cells	0.026 (0.021); 0.024 (0-0.115)	0.003 (0.007); 0 (0-0.075)	0.008 (0.002)	7.97E-06	0.024 (0.021); 0.024 (0-0.115)	0.026 (0.022); 0.024 (0-0.115)	0.001 (0.004)	0.74	-0.004 (0.010)	0.67
Monocytes	0.070 (0.039); 0.070 (0-0.190)	0.083 (0.027); 0.080 (0.009-0.167)	-0.044 (0.004)	3.75E-23	0.054 (0.037); 0.070 (0-0.190)	0.072 (0.038); 0.074 (0-0.190)	-0.014 (0.006)	0.026	-0.063 (0.017)	2.82E-04
Granulocytes	0.593 (0.192); 0.648 (0-0.879)	0.655 (0.084); 0.660 (0.301-0.836)	-0.163 (0.013)	8.10E-32	0.472 (0.239); 0.649 (0-0.879)	0.614 (0.179); 0.655 (0-0.879)	-0.106 (0.031)	8.71E-04	-0.485 (0.080)	9.81E-09
nRBCs	0.116 (0.228); 0 (0-1.00)	0.005 (0.018); 0 (0-0.220)	0.299 (0.015)	4.45E-65	0.221 (0.283); 0 (0-1.00)	0.102 (0.218); 0 (0-1.00)	0.080 (0.039)	0.045	0.433 (0.104)	5.03E-05

P-values and coefficients calculated using linear regression, testing each blood cell type separately as the dependent variable, with DS status or *GATA1* mutation as the independent variable, and including plate, sex, blood collection age, gestational age, birth weight, and ten ancestry-related principal components from EPISTRUCTURE as covariates.

* P-values and coefficients calculated in linear regression models including *GATA1* mutations treated as a binary variable (presence/absence).

** P-values and coefficients calculated in linear regression models including *GATA1* mutation variant allele fraction (VAF) as a continuous variable.

P-values were not adjusted for multiple comparisons.

Abbreviations: NK: natural killer, nRBC: nucleated red blood cell, SD: standard deviation, SE: standard error.

Supplementary Table 3 – Direction of CpG methylation changes in Down syndrome on Hsa21 and non-Hsa21 chromosomes.

		Total N CpGs	Mean Beta in DS EWAS	N hypermethylated	N hypomethylated	P-value*
**All CpGs**	**Hsa21**	7,351	-0.0052	2,926 (39.8%)	4,425 (60.2%)	
	**Non-Hsa21**	644,421	0.00037	288,199 (44.7%)	356,222 (55.3%)	<.0001
**CpG_islands**	**Hsa21**	1,365	-0.00047	556 (40.7%)	809 (59.3%)	
	**Non-Hsa21**	127,257	-0.00053	44,820 (35.2%)	82,437 (64.8%)	<.0001
**CpG_shelves**	**Hsa21**	565	-0.0077	204 (36.1%)	361 (63.9%)	
	**Non-Hsa21**	44,465	-0.0000053	21,091 (47.4%)	23,374 (52.6%)	<.0001
**CpG_shores**	**Hsa21**	1,160	-0.005	434 (37.4%)	726 (62.6%)	
	**Non-Hsa21**	118,307	0.00047	56,774 (48.0%)	61,533 (52.0%)	<.0001
**inter-CGI**	**Hsa21**	4,261	-0.0064	1,732 (40.6%)	2,529 (59.4%)	
	**Non-Hsa21**	354,392	0.00071	165,514 (46.7%)	188,878 (53.3%)	<.0001

* P-values calculated using Chi-square tests.

Positions of CpGs (CpG_islands, CpG_shelves, CpG_shores, inter-CGI) were determined using the annotation database from the "IlluminaHumanMethylationEPICanno.ilm10b4.hg19" package in R.

Abbreviations: CGI: CpG islands.

Supplementary Table 4 – Primer sequences used for targeted sequencing of *GATA1* mutations in newborns with Down syndrome.

Assay Name	Chr	Start (bp)	End (bp)	Forward primer (incl. adapters)*	Reverse primer (incl. adapters)*	Amplicon length (bp)	GC content (%)
GATA1_1	X	48649426	48649624	ACACTGACGACATGGTTCTACAAGGAAGAGGAGCAGGTGAAAG	TACGGTAGCAGAGACTTGGTCTAGAGGGGAAGAAAACCCCTGATT	199	59
GATA1_2	X	48649432	48649624	ACACTGACGACATGGTTCTACAaggagcaggTGAAAGGATGTG	TACGGTAGCAGAGACTTGGTCTAGAGGGGAAGAAAACCCCTGATT	193	59
GATA1_3	X	48649465	48649664	ACACTGACGACATGGTTCTACATTCTGTGTCTGAGGACCCCTT	TACGGTAGCAGAGACTTGGTCTGGGCAGTGGAGGAAGCTG	200	62
GATA1_4	X	48649545	48649712	ACACTGACGACATGGTTCTACAGGACCTCAGAGCCCCTCC	TACGGTAGCAGAGACTTGGTCTCGTCCCTGTAGTAGGCCAGT	168	64
GATA1_5	X	48649601	48649798	ACACTGACGACATGGTTCTACAGAATCAGGGGTTTTCTTCCCCTC	TACGGTAGCAGAGACTTGGTCTGATCTCCATGGCAACCCCAAC	198	61
GATA1_6	X	48650186	48650366	ACACTGACGACATGGTTCTACATGACGTGCGCTGACCCTA	TACGGTAGCAGAGACTTGGTCTGGTGGGACACACAGTTGAGG	181	59

Genomic positions correspond to human genome build hg19.

* Sequencing adapter sequences are highlighted by underlining in each primer pair.

Supplementary Figure 1 – Confirmation of trisomy 21 in newborns with Down syndrome by copy number variation (CNV) analysis of genome-wide DNA methylation data. Copy number variation across autosomal chromosomes was predicted from Illumina Infinium EPICmethylation Beadchip array data in DS (teal) and non-DS subjects (red) using the "conumee" package in R. Increased copy number (red) across chromosome 21 is clearly observed among DS (N=196) but not non-DS newborns (N=439, from which a DS outlier has already been removed). Two potential DS outliers for chromosome 21 copy number are examined in Supplementary Figure 2.

Figure S1

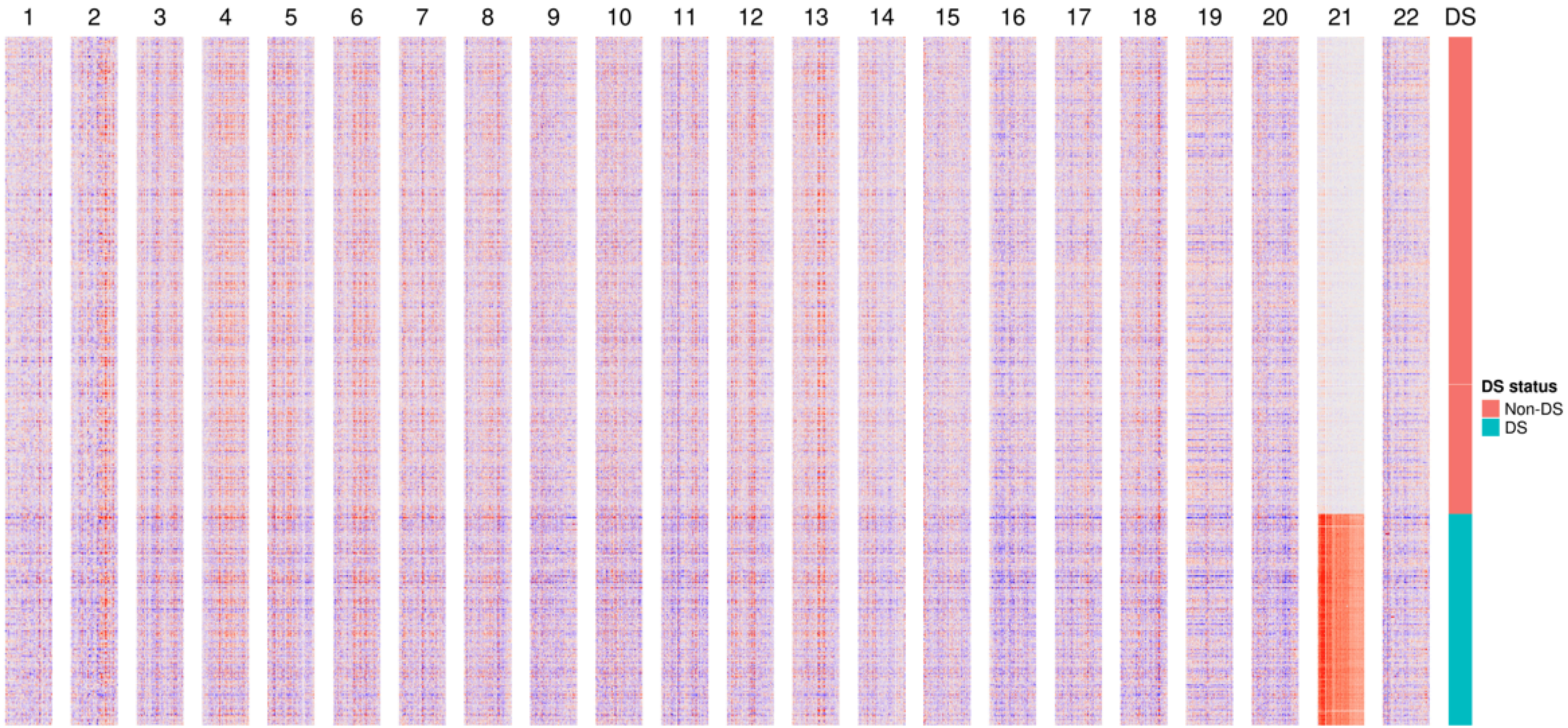
1
2
3
4
5
6
7
8
9
10
11
12
13
14
15
16
17
18
19
20
21
22
DS
DS status
Non-DS
DS

Supplementary Figure 2 – Two newborns with Down syndrome with apparent mosaic trisomy 21. Two newborns with DS according to data reported in the California Biobank Program were found to cluster among the non-DS newborns in PCA, t-SNE, and hierarchical clustering plots (**Figures 1 and 2**). The R package "conumee" was used to generate copy number variation (CNV) plots for all samples to check trisomy 21 status, with 20 randomly selected non-DS samples used to construct a CNV reference. Both DS newborns that clustered with non-DS newborns appear to have increased copy number on chromosome 21 relative to other chromosomes (panels **b** and **c**), but lower than the increase found in other DS samples (e.g., panel **a**). The boxplot in panel **d** shows the mean log_2 ratio on chromosome 21 calculated across 317 bins generated by conumee, for non-DS (N=439) and DS (N=196) subjects, with the two likely mosaic DS newborns highlighted at a lower than expected mean log_2 ratio. In the boxplot, the center line represents the median, the upper and lower bounds of the box represent the interquartile range (IQR, the range between the 25th and 75th percentiles), and whiskers extend to the highest and lowest values within 1.5 times the IQR.

Figure S2

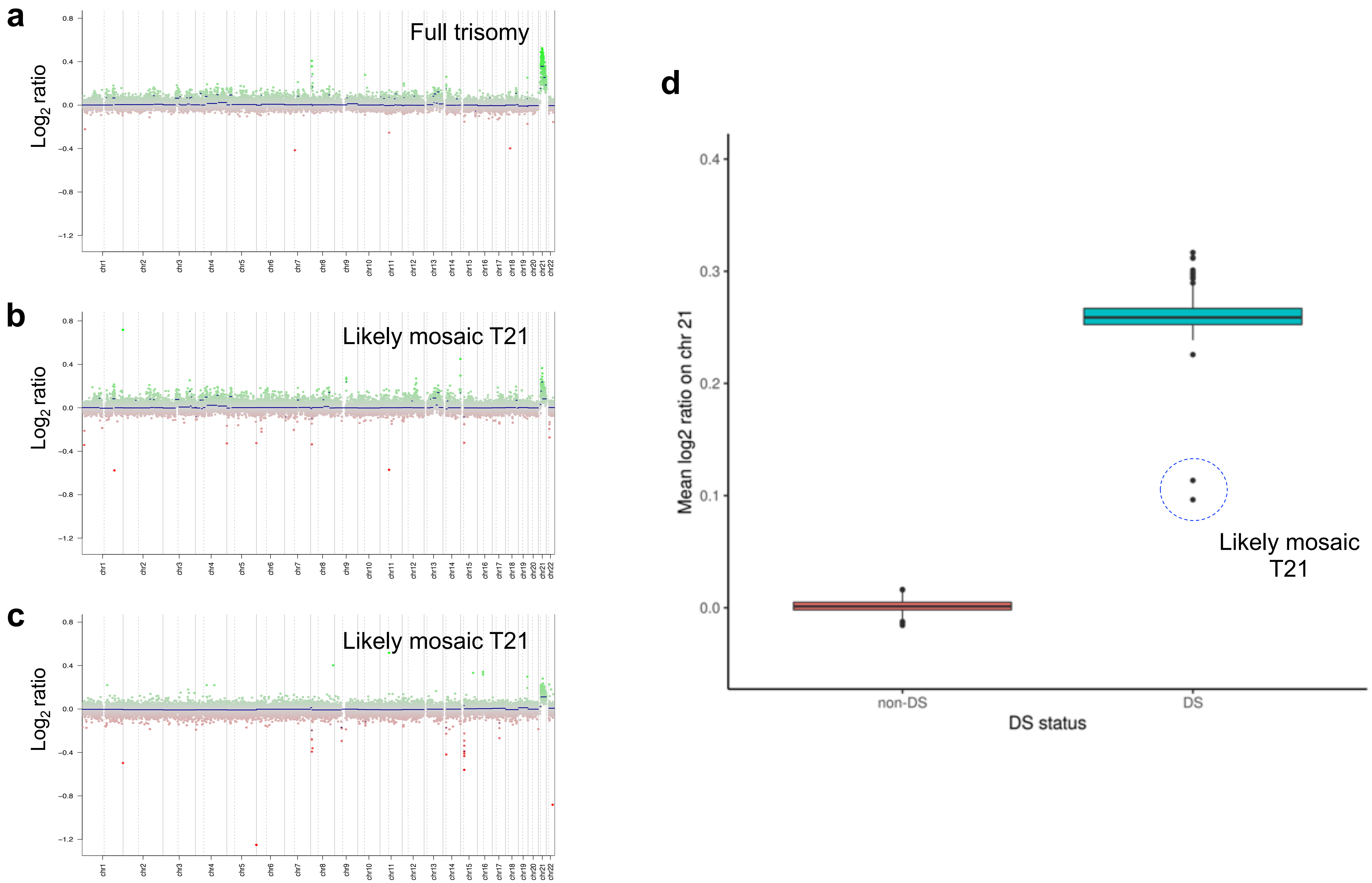
a
Full trisomy
b
Likely mosaic T21
c
Likely mosaic T21
Log2 ratio
0.8
0.4
0.0
−0.4
−0.8
−1.2
chr1
chr2
chr3
chr4
chr5
chr6
chr7
chr8
chr9
chr10
chr11
chr12
chr13
chr14
chr15
chr16
chr17
chr18
chr19
chr20
chr21
chr22
d
Mean log2 ratio on chr 21
0.4
0.3
0.2
0.1
0.0
Likely mosaic T21
non-DS
DS
DS status

Supplementary Figure 3 – Scatter plot displaying the variant allele frequency (VAF) of somatic *GATA1* mutations versus their effect on protein coding, in 35 variants detected among 184 newborns with Down syndrome. Somatic *GATA1* mutations with predicted functional effects (missense, frameshift insertion/deletion, splicing, nonsense) had significantly higher VAF than noncoding mutations (P=.0085, two-sided Wilcoxon rank-sum test). Two mutations – one missense and one noncoding – were detected on the same strand (red asterisks); following removal of these mutations, the difference in VAF between noncoding and functional mutations became more significant (P=.0034, two-sided Wilcoxon rank-sum test).

Figure S3

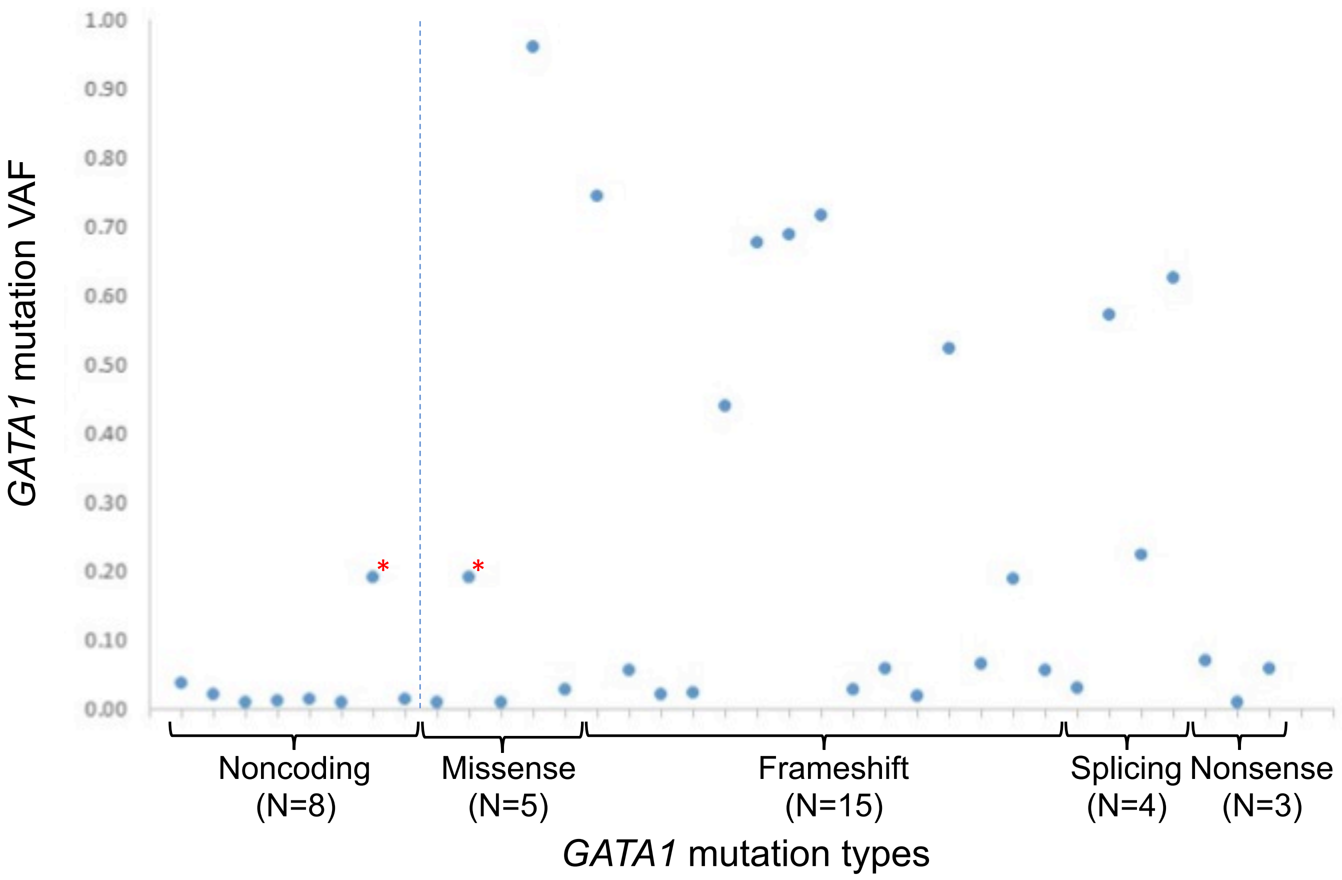

* Noncoding and coding variants on same strand

***P* = 0.0034** following removal of these two variants, two-tailed Wilcoxon rank-sum test

Supplementary Figure 4 – Blood cell proportions deconvoluted from DNA methylation data in newborns with and without Down syndrome, and by *GATA1* mutation status.

Relative blood cell type proportions at birth in 196 DS (teal/blue) and 439 non-DS newborns (red) were derived from genome-wide DNA methylation array data from newborn dried bloodspots using the IDOL deconvolution algorithm. Blood cell proportions were deconvoluted for $CD8^+$ T-cells, $CD4^+$ T-cells, natural killer (NK) cells, B-cells, monocytes, granulocytes, and nucleated red blood cells (nRBCs). Deconvoluted blood cell proportions were visualized by boxplots generated in R. Panel **a** displays blood cell proportions in overall DS versus non-DS newborns, with strongly significant differences found across all comparisons in separate linear regression models, except for $CD8^+$ T-cells ($CD8^+$ T-cells, P=0.073; $CD4^+$ T-cells, P=2.26×10^{-53}; NK cells, P=7.97×10^{-6}; B-cells, P=3.48×10^{-28}; monocytes, P=3.75×10^{-23}; granulocytes, P=8.10×10^{-32}; nRBCs, P=4.45×10^{-65}). Panel **b** displays blood cell proportions in DS newborns stratified into *GATA1* mutation-positive (blue, N=30) and wildtype (teal, N=154), and in non-DS newborns (red, N=439). Significant differences between *GATA1* mutant and wildtype DS newborns were found in separate linear regression models for $CD4^+$ T-cells (P=2.17×10^{-5}), monocytes (P=0.026), granulocytes (P=8.71×10^{-4}), and nRBCs (P=0.045), but not for other cell types ($CD8^+$ T-cells, P=0.24; NK cells, P=0.74; B-cells, P=0.13).

In the boxplots, the center line represents the median, the upper and lower bounds of the box represent the interquartile range (IQR, the range between the 25th and 75th percentiles), and whiskers extend to the highest and lowest values within 1.5 times the IQR.

P-values were calculated by linear regression tests with each cell type as the dependent variable, and adjusting for plate, sex, age at bloodspot collection, gestational age, birthweight, and ten EPISTRUCTURE PCs. Significant P-values denoted as: *=P<.05, **=P<5×10^{-3}, ***=P<5×10^{-5}.

Figure S4

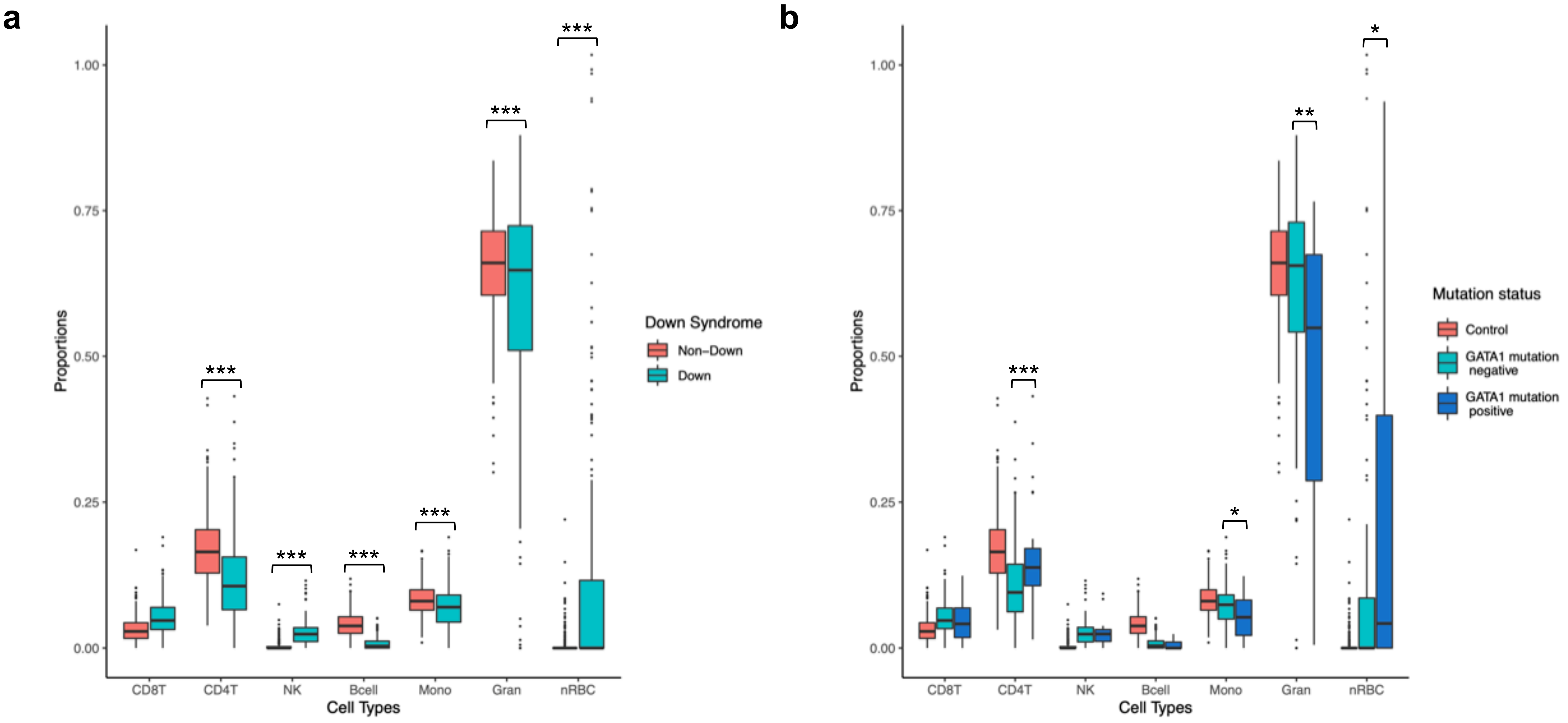
a
b
1.00
0.75
0.50
0.25
0.00
Proportions
***
***
***
***
***
***
CD8T
CD4T
NK
Bcell
Mono
Gran
nRBC
Cell Types
Down Syndrome
Non–Down
Down
*
**
*
***
Mutation status
Control
GATA1 mutation negative
GATA1 mutation positive

Supplementary Figure 5 – Blood cell proportions deconvoluted from DNA methylation data in newborns with and without Down syndrome in Latinos and non-Latino whites. Relative blood cell type proportions at birth in DS (teal) and non-DS subjects (red) were derived from genome-wide DNA methylation array data from newborn dried bloodspots using the IDOL deconvolution algorithm. Blood cell proportions were deconvoluted for $CD8^+$ T-cells, $CD4^+$ T-cells, natural killer (NK) cells, B-cells, monocytes, granulocytes, and nucleated red blood cells (nRBCs). Deconvoluted blood cell proportions were visualized by boxplots generated in R. Panel **a** displays blood cell proportions in Latino DS (N=104) versus non-DS (N=253) newborns ($CD8^+$ T-cells, P=0.98; $CD4^+$ T-cells, P=1.46x10^{-22}; NK cells, P=2.11x10^{-3}; B-cells, P=4.52x10^{-13}; monocytes, P=2.28x10^{-12}; granulocytes, P=2.68x10^{-15}; nRBCs, P=4.40x10^{-31}). Panel **b** displays proportions in non-Latino white DS (N=54) versus non-DS (N=124) newborns ($CD8^+$ T-cells, P=0.034; $CD4^+$ T-cells, P=7.26x10^{-25}; NK cells, P=4.29x10^{-3}; B-cells, P=3.28x10^{-7}; monocytes, P=1.33x10^{-6}; granulocytes, P=4.93x10^{-12}; nRBCs, P=5.05x10^{-30}). In both analyses, strongly significant differences were found across all comparisons in separate linear regression models, except for $CD8^+$ T-cells. In the boxplots, the center line represents the median, the upper and lower bounds of the box represent the interquartile range (IQR, the range between the 25th and 75th percentiles), and whiskers extend to the highest and lowest values within 1.5 times the IQR.

P-values were calculated by linear regression tests with each cell type as the dependent variable, and adjusting for plate, sex, age at bloodspot collection, gestational age, birthweight, and ten EPISTRUCTURE PCs. Significant P-values denoted as: *=P<.05, **=P<5x10^{-3}, ***=P<5x10^{-5}.

Figure S5

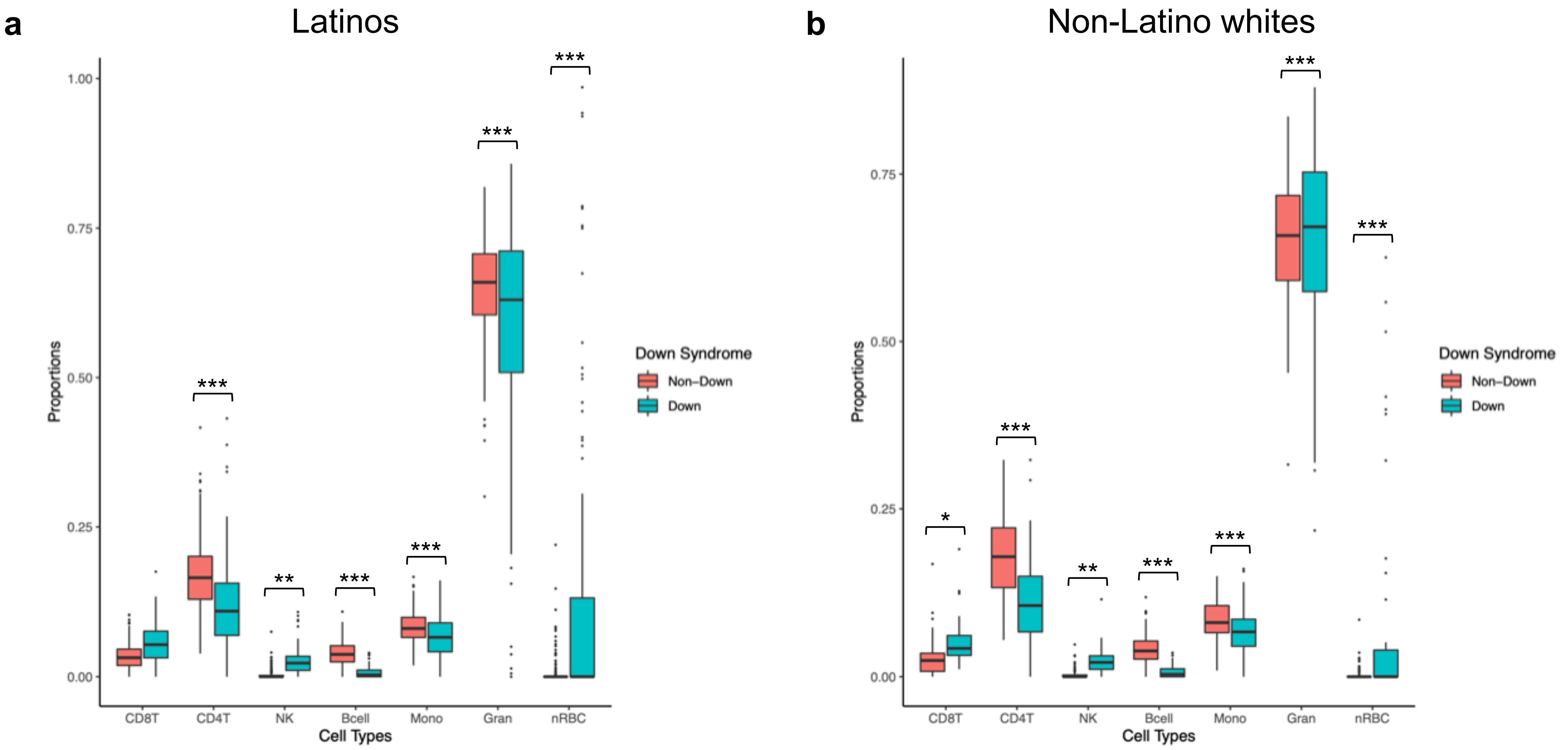
a
Latinos
b
Non-Latino whites
Proportions
1.00
0.75
0.50
0.25
0.00
***
**
*
CD8T
CD4T
NK
Bcell
Mono
Gran
nRBC
Cell Types
Down Syndrome
Non-Down
Down

Supplementary Figure 6 – Scatter plot displaying the proportion of nucleated red blood cells (nRBC) versus *GATA1* mutation variant allele frequency (VAF) in 184 newborns with Down syndrome. nRBC proportions were deconvoluted from DNA methylation array data using the IDOL algorithm. Targeted sequencing revealed 30 of 184 DS newborns harbored somatic *GATA1* mutations. Linear regression was performed to assess the relationship between *GATA1* mutation VAF and nRBC proportions, adjusting for plate, sex, age at bloodspot collection, gestational age, birthweight, and ten EPISTRUCTURE PCs, and found significant positive association ($P=5.0x10^{-5}$).

Figure S6

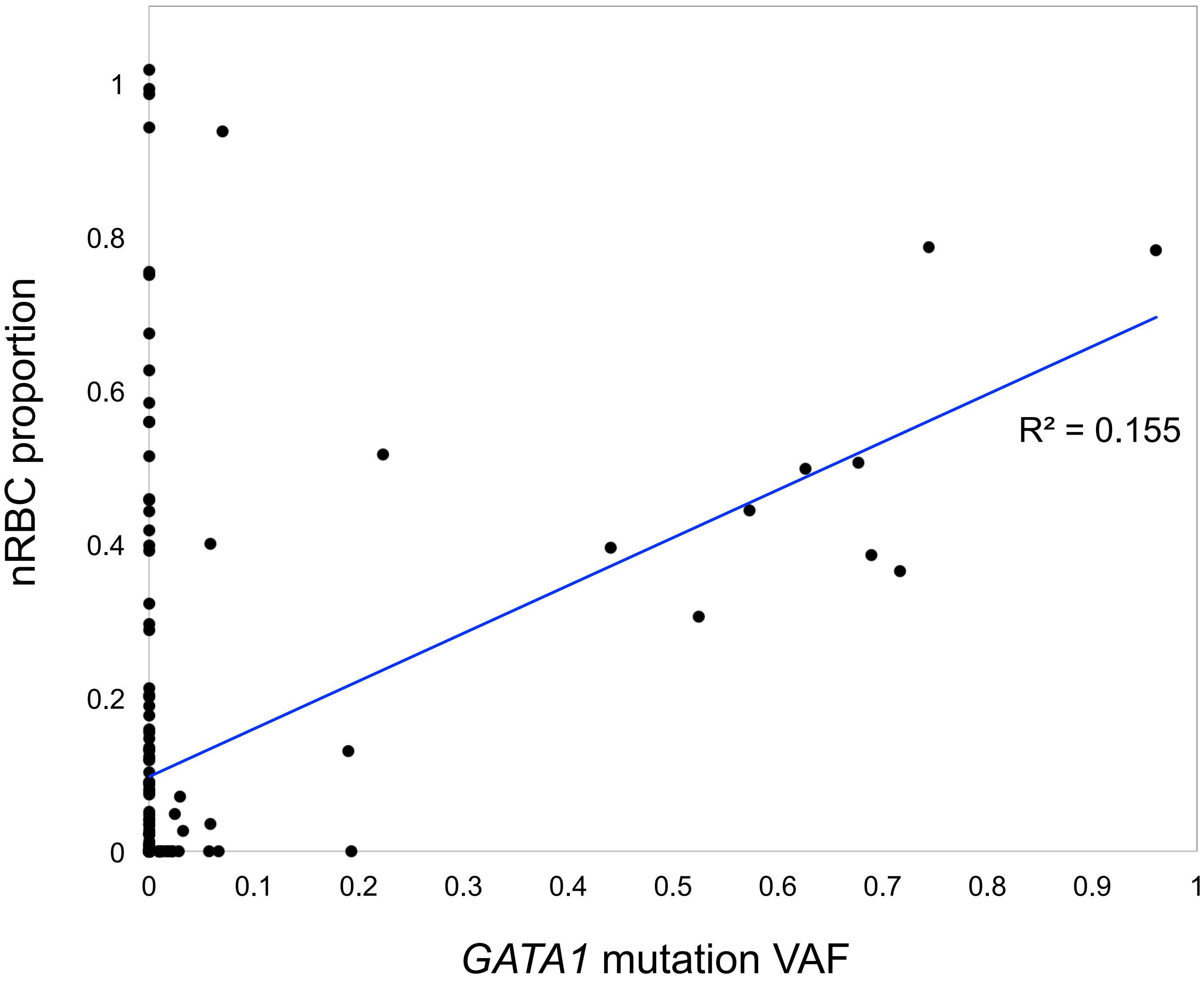

nRBC proportion
1
0.8
0.6
0.4
0.2
0
R² = 0.155
0
0.1
0.2
0.3
0.4
0.5
0.6
0.7
0.8
0.9
1
GATA1 mutation VAF

Supplementary Figure 7 – Significant hypermethylation in Down syndrome at *RUNX1* on chromosome 21, which is specific to the P2 promoter.

a: Manhattan plot displaying the $-\log_{10}$(P)-values of CpGs on chromosome 21, derived from the multi-ethnic EWAS of DS. Points above zero correspond to CpGs that were hypermethylated in DS versus non-DS newborns, whereas points below zero correspond to hypomethylated CpGs in DS. P-values were derived from linear regression tests adjusting for sex, plate, the first ten EPISTRUCTURE PCs to control for genetic ancestry, and the first ten ReFACTor PCs to control for cell mixture. The dotted lines correspond to the threshold for epigenome-wide significance ($P=7.67 \times 10^{-8}$) after Bonferroni correction for multiple testing, based on 651,773 CpGs. The strong association peak at *RUNX1* is highlighted.

b: Plot displaying the DS EWAS results for 122 CpG probes across the *RUNX1* gene (chr21: 36163427-36423674, hg19). Chromosome 21 positions of the proximal P2 (chr21:36260988-36261987) and distal P1 promoters of *RUNX1* were retrieved using the UCSC Genome Browser Table Browser, for the transcription start site of each relevant transcript minus 1,000bp. Significantly hypermethylated DS-associated CpGs were identified at the P2 promoter, but not the P1 promoter region. Two DS-associated, hypermethylated DMRs (DMR1, DMR2) were detected at the P2 promoter, and one hypomethylated DMR (DMR3) was identified at the P1 promoter.

Figure S7

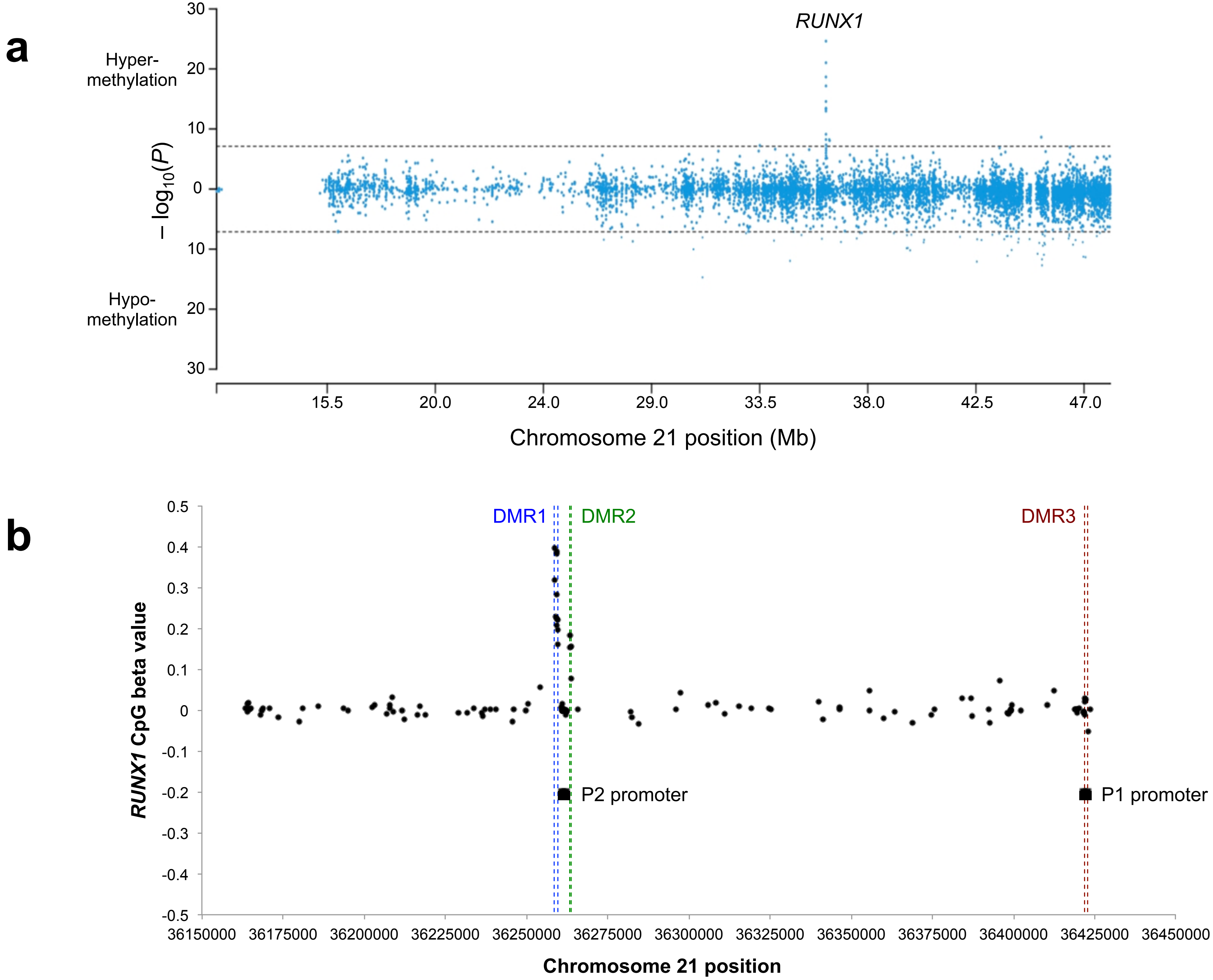
a
RUNX1
Hyper-
methylation
Hypo-
methylation
30
20
10
0
10
20
30
− log10(P)
15.5
20.0
24.0
29.0
33.5
38.0
42.5
47.0
Chromosome 21 position (Mb)
b
DMR1
DMR2
DMR3
P2 promoter
P1 promoter
0.5
0.4
0.3
0.2
0.1
0
-0.1
-0.2
-0.3
-0.4
-0.5
RUNX1 CpG beta value
36150000
36175000
36200000
36225000
36250000
36275000
36300000
36325000
36350000
36375000
36400000
36425000
36450000
Chromosome 21 position

Supplementary Figure 8 – Enrichment of Down syndrome-associated CpGs in epigenomic features by hyper- and hypomethylation status. Bar charts representing the proportions of epigenome-wide significant and non-significant CpGs that overlapped genomic and epigenomic features including: promoters, exons, introns, intergenic regions, CpG islands, shores, and shelves. Hypomethylated CpGs (green) were significantly underrepresented at promoter regions ($P=6.14x10^{-4}$) and at CpG islands ($P=3.59x10^{-10}$) but significantly enriched in intergenic regions ($P=1.56x10^{-6}$) compared to non-significant CpGs (chi-square test). Significant P-values denoted as: $**=P<5x10^{-3}$, $***=P<5x10^{-5}$.

Figure S8

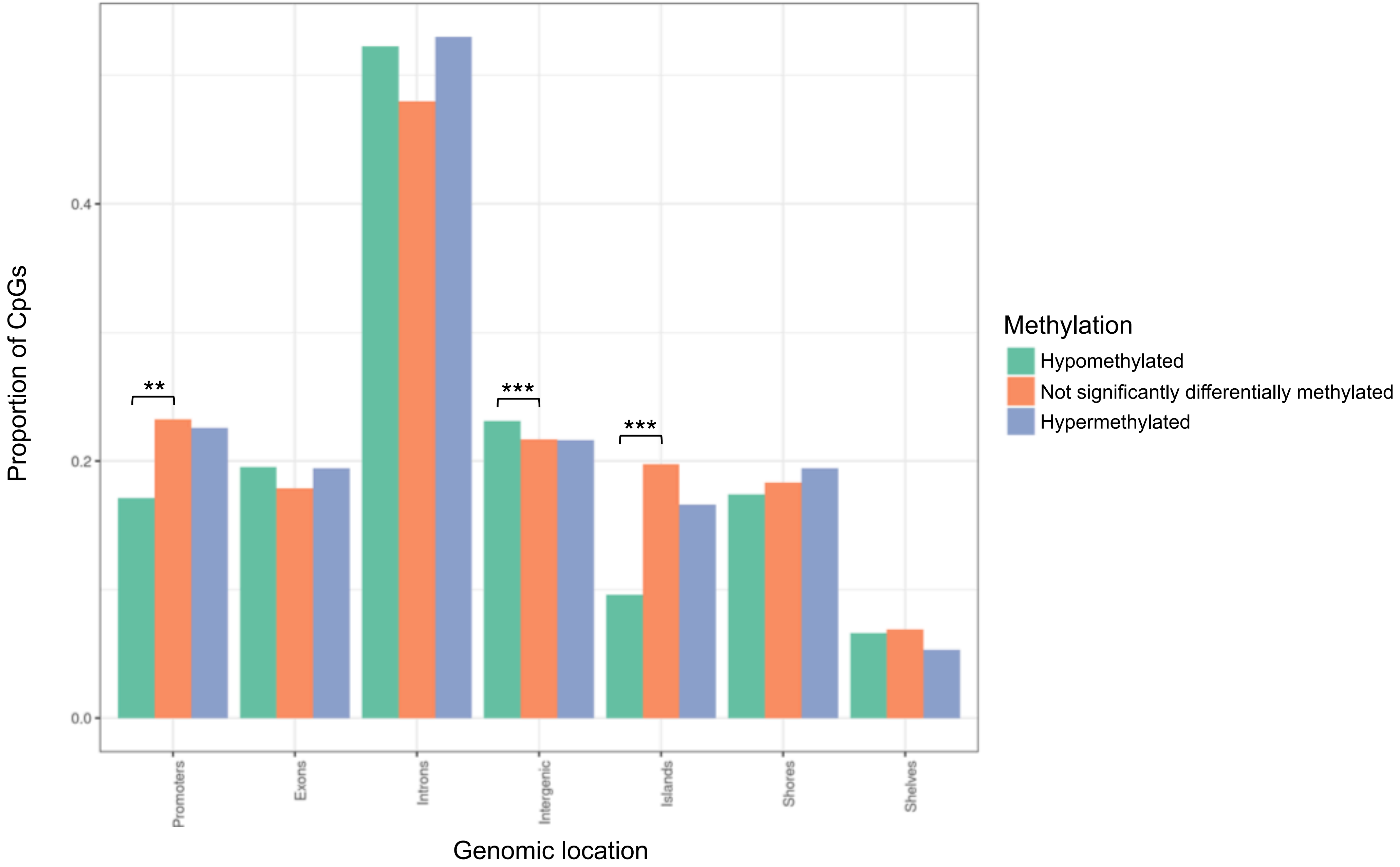
Proportion of CpGs
0.0
0.2
0.4
**
***
***
Promoters
Exons
Introns
Intergenic
Islands
Shores
Shelves
Genomic location
Methylation
Hypomethylated
Not significantly differentially methylated
Hypermethylated

Supplementary Figure 9 – Proportion of Down syndrome-associated differentially methylated regions (DMRs) across autosomal chromosomes. DMRs were identified on all chromosomes, with a particularly high proportion (11.2%) on chromosome 21, as well as chromosomes 1, 6, 11, 12, 16, 17, and 19.

Figure S9

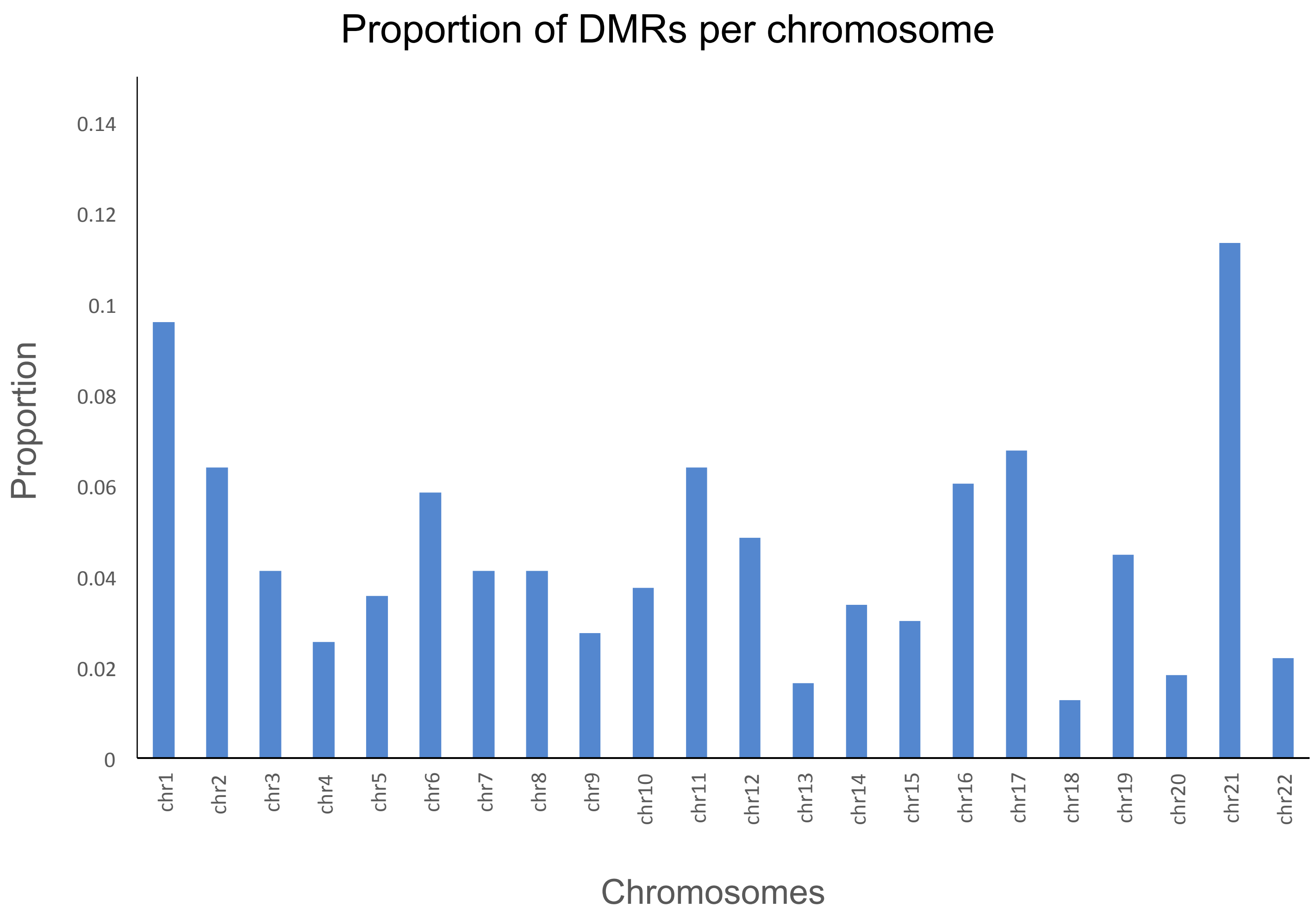
Proportion of DMRs per chromosome
Proportion
0.14
0.12
0.1
0.08
0.06
0.04
0.02
0
chr1
chr2
chr3
chr4
chr5
chr6
chr7
chr8
chr9
chr10
chr11
chr12
chr13
chr14
chr15
chr16
chr17
chr18
chr19
chr20
chr21
chr22
Chromosomes

Supplementary Figure 10 – Expression of FLI1 protein transcript variants across blood cell types in the BLUEPRINT Blood Atlas. Plots displaying *FLI1* transcript variant expression across 27 blood cell types, calculated as mean expression in each cell type (**a**) and as the expression in each cell type relative to total expression across all cell types (**b**). Data were downloaded from the BLUEPRINT Consortium Blood Atlas (https://blueprint.haem.cam.ac.uk/bloodatlas/), which includes expression data from whole transcriptome RNA sequencing in 27 cell types from cord blood (Cb) and adult peripheral venous blood (Vb) (https://blueprint.haem.cam.ac.uk/bloodatlas/samples.html), and are displayed for the 4 main protein coding *FLI1* transcripts. The DS-associated DMR at *FLI1* overlapped the promoter for transcript variant 4, which was mainly expressed in cord blood megakaryocyte cells, a pattern not seen in other transcript variants.

Figure S10

a **b**

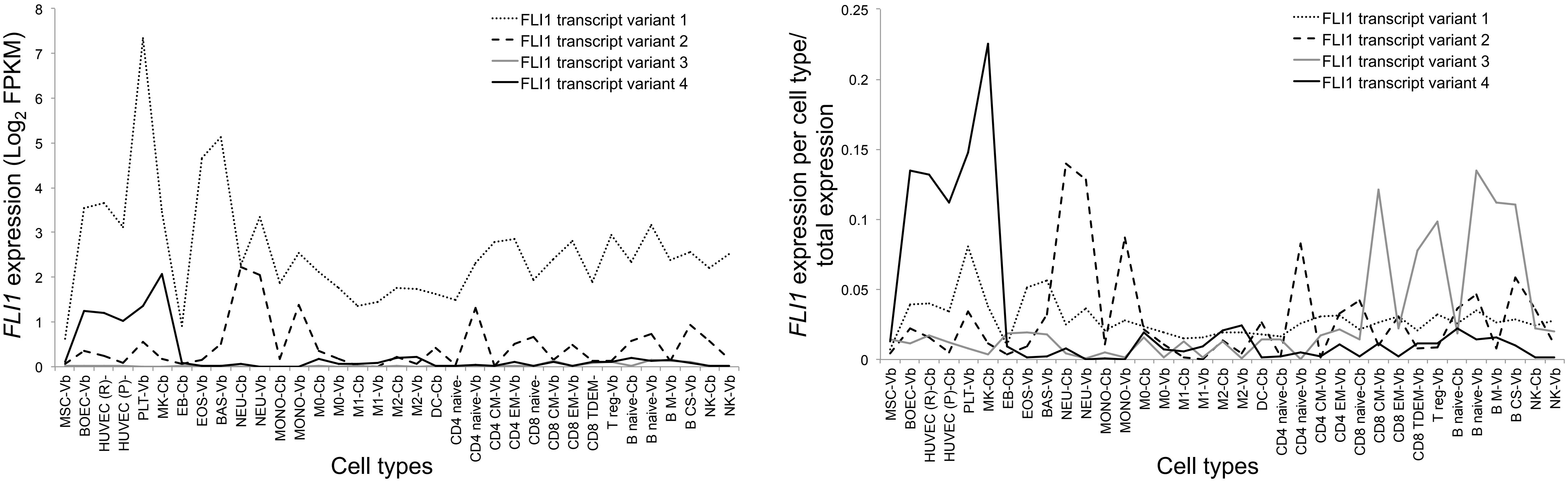

Vb: adult venous blood; **Cb:** cord blood.

MSC:mesenchymal stem cell of the bone marrow; **BOEC**:blood outgrowth endothelial cells; **HUVEC (R)**:human umbilical vein endothelial cells (resting); **HUVEC (P)**:human umbilical vein endothelial cells (proliferating); **PLT**:platelets; **MK**:CD34-negative, CD41-positive, CD42-positive megakaryocyte cell; **EB**:erythroblast; **EOS**:eosinophil; **BAS**:basophil; **NEU**:mature neutrophil; **MONO**:CD14-positive, CD-16 negative classical monocyte; **M0**:macrophage; **M1**:inflammatory macrophage; **M2**:alternatively activated macrophage; **DC**:conventional dendritic cell; **CD4 naive**:CD4-positive, alpha-beta T cell; **CD4 CM**:central memory CD4-positive, alpha-beta T cell; **CD4 EM**:effector memory CD4-positive, alpha-beta T cell; **CD8 naive**:CD8-positive, alpha-beta T cell; **CD8 CM**:central memory CD8-positive, alpha-beta T cell; **CD8 EM**:effector memory CD8-positive, alpha-beta T cell; **CD8 TDEM**:terminally differentiated effector memory CD8-positive, alpha-beta T cell; **T reg**:regulatory T cell; **B naive**:CD38-negative naive B cell; **B M**:memory B cell; **B CS**:class switched memory B cell; **NK**:cytotoxic CD56-dim natural killer cell.

Supplementary Figure 11 – Down syndrome-associated DMRs with large differences in DNA methylation. DS-associated DMRs overlapping regulatory regions in *CPT1B* (**a**), *PRDM8* (**b**), *ASB3* (**c**), and *CMYA5* (**d**). These DMRs all include at least 10 CpG probes with a mean beta difference >0.10.

Figure S11a

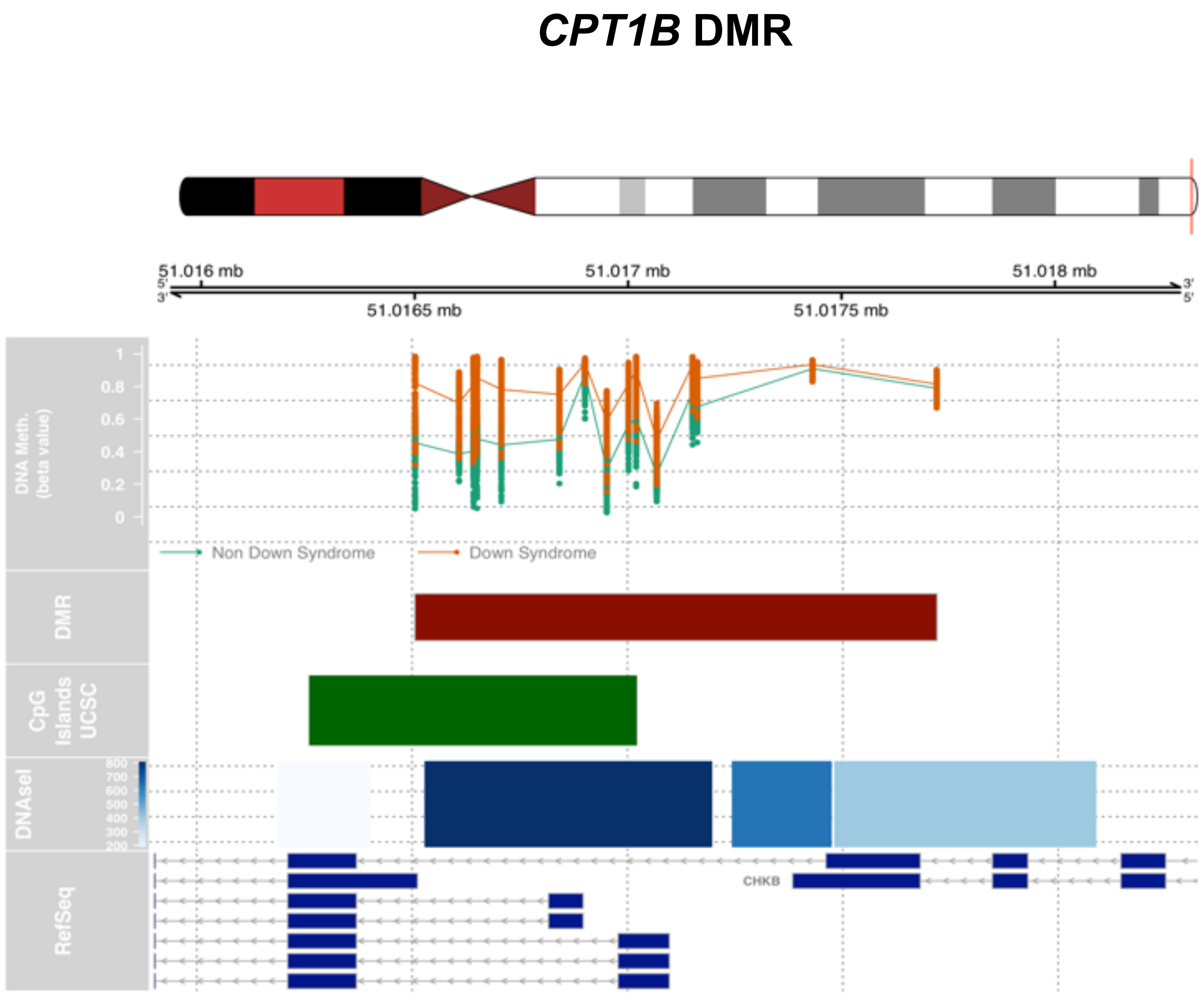
CPT1B DMR
51.016 mb
51.017 mb
51.018 mb
51.0165 mb
51.0175 mb
DNA Meth. (beta value)
1
0.8
0.6
0.4
0.2
0
Non Down Syndrome
Down Syndrome
DMR
CpG Islands UCSC
DNAseI
800
700
600
500
400
300
200
RefSeq
CHKB

Figure S11b

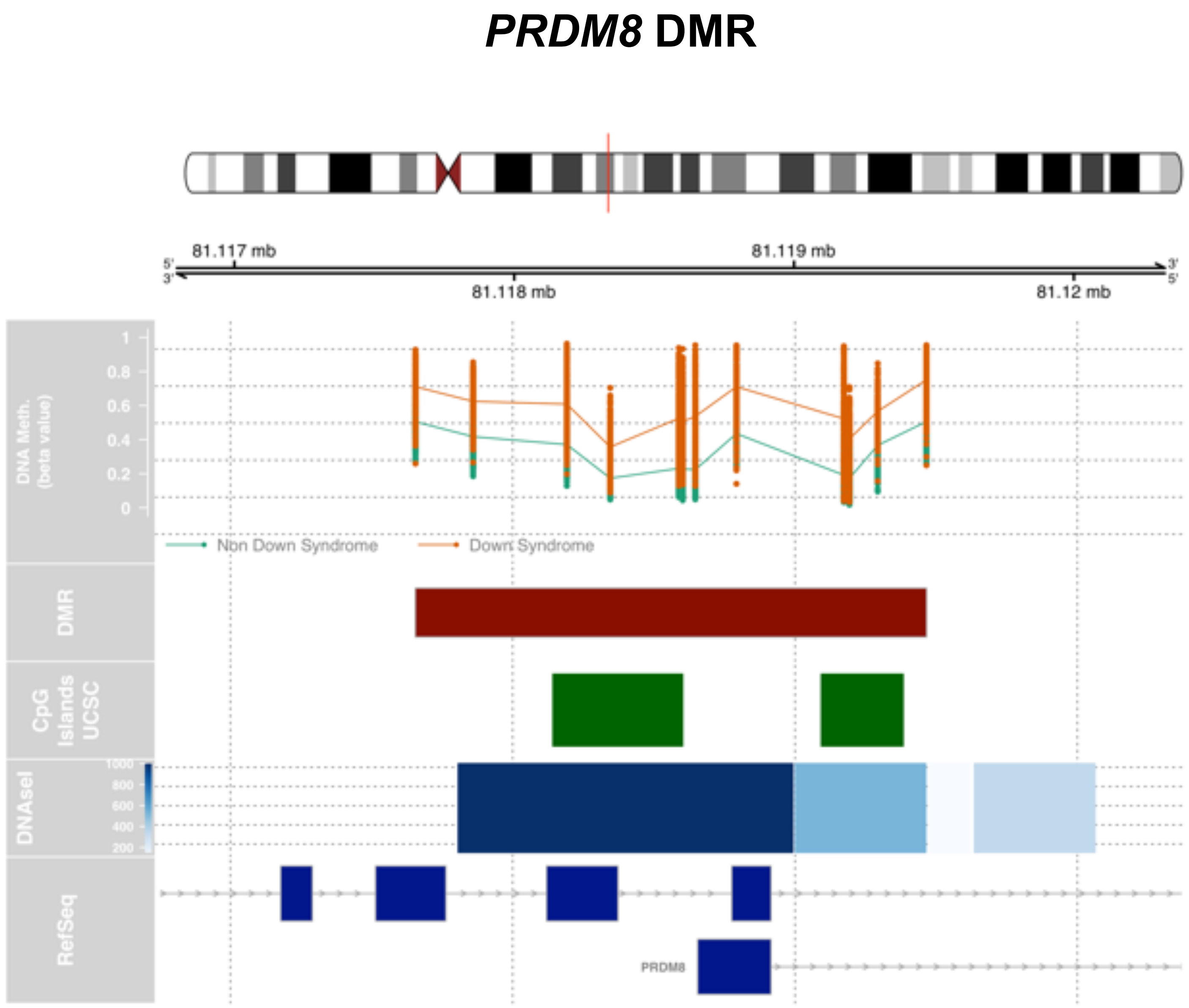

PRDM8 DMR
81.117 mb
81.119 mb
81.118 mb
81.12 mb
DNA Meth. (beta value)
1
0.8
0.6
0.4
0.2
0
Non Down Syndrome
Down Syndrome
DMR
CpG Islands UCSC
DNAseI
1000
800
600
400
200
RefSeq
PRDM8

Figure S11c

ASB3 DMR

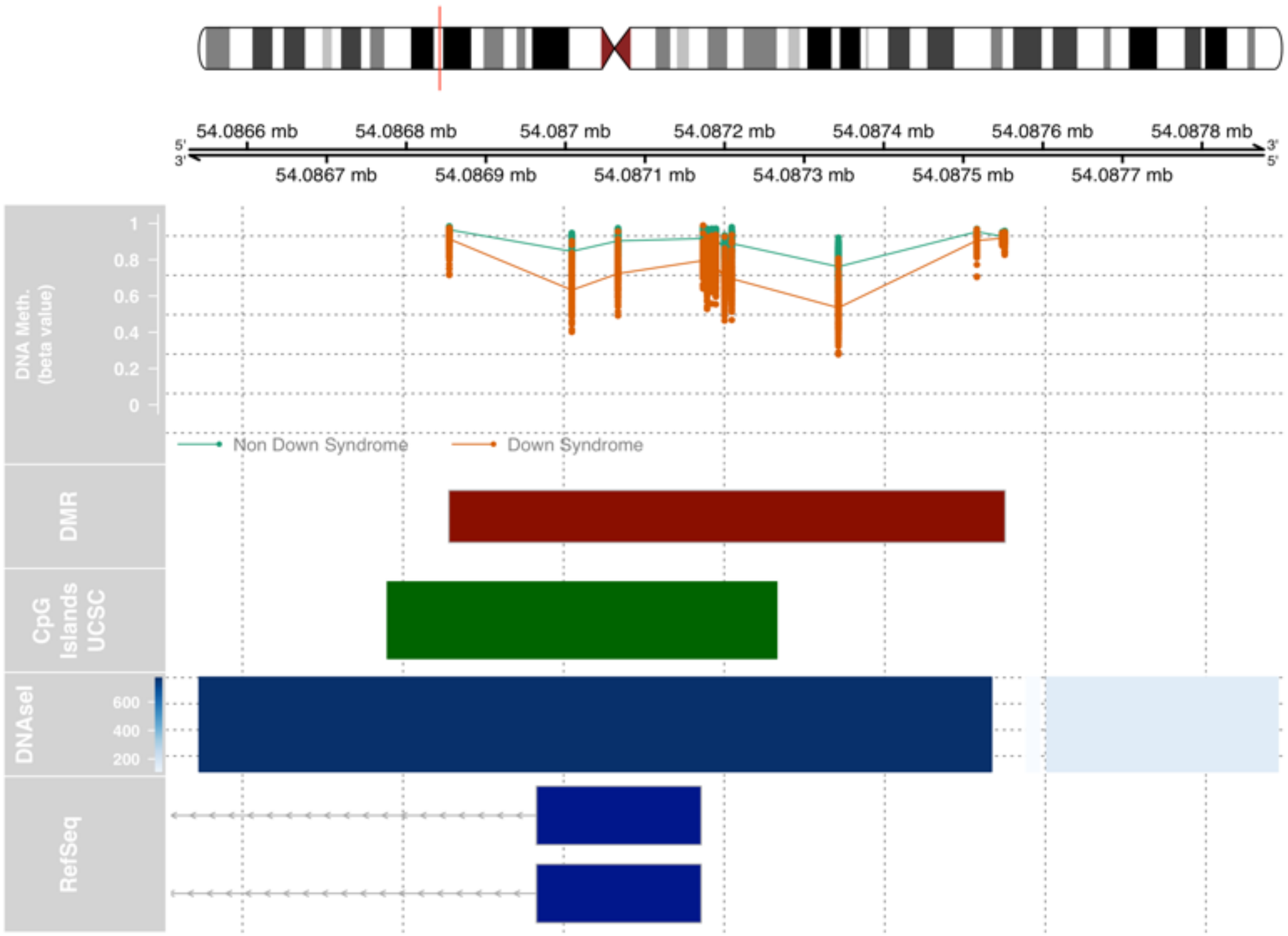

Figure S11d

CMYA5 DMR

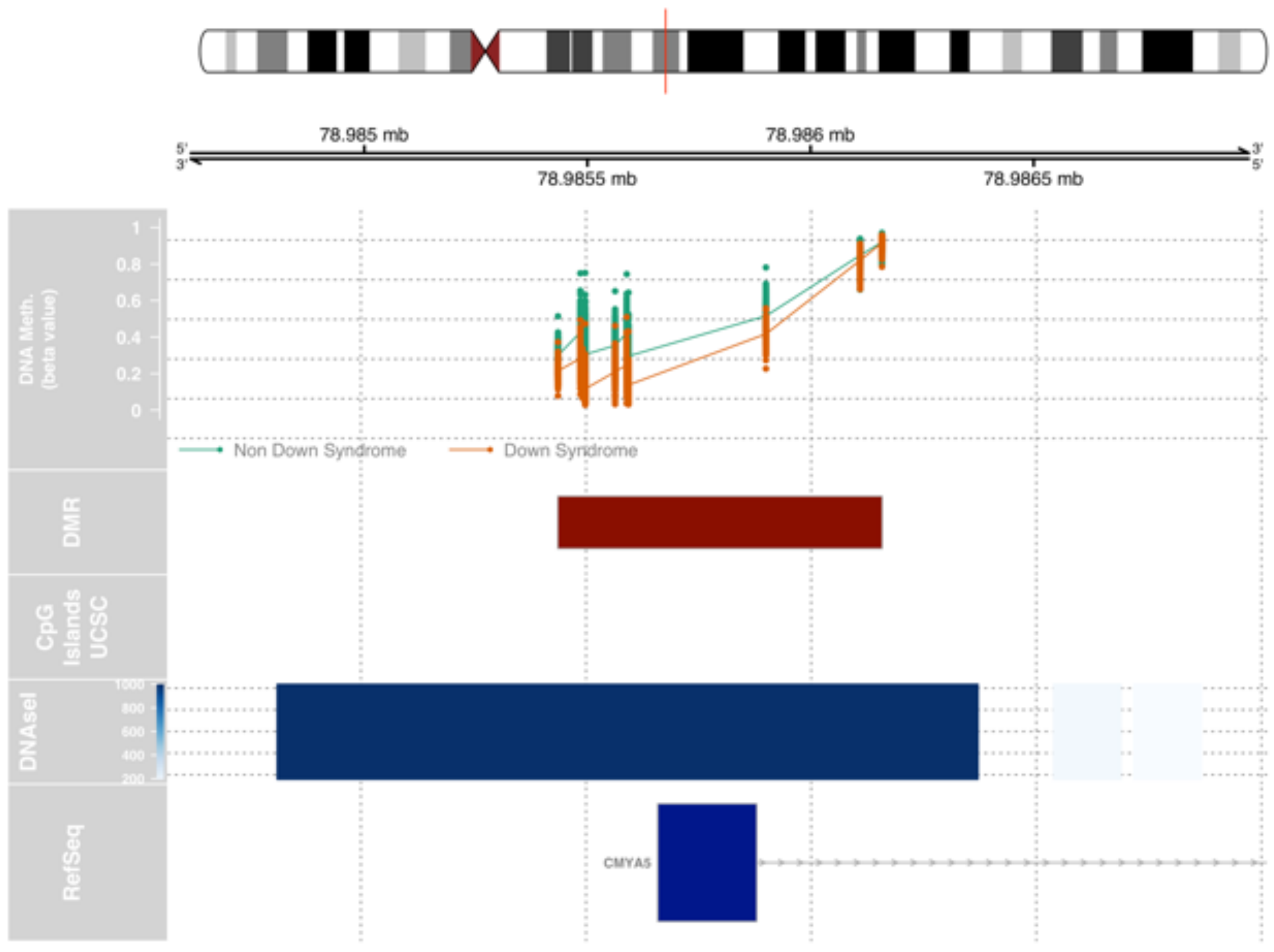

Supplementary Figure 12 – *RUNX1* promoter DMR mean DNA methylation levels in newborns with and without Down syndrome, and by *GATA1* mutation status. Boxplots displaying the mean DNA methylation beta values across 11 CpGs in a *RUNX1* promoter DMR: (**a**) among non-DS newborns (N=439) versus DS newborns (N=196), and (**b**) among *GATA1* mutation-positive DS newborns (N=30) versus mutation wildtype (wt) newborns (N=154). In the boxplots, the center line represents the median, the upper and lower bounds of the box represent the interquartile range (IQR, the range between the 25th and 75th percentiles), and whiskers extend to the highest and lowest values within 1.5 times the IQR.

Figure S12

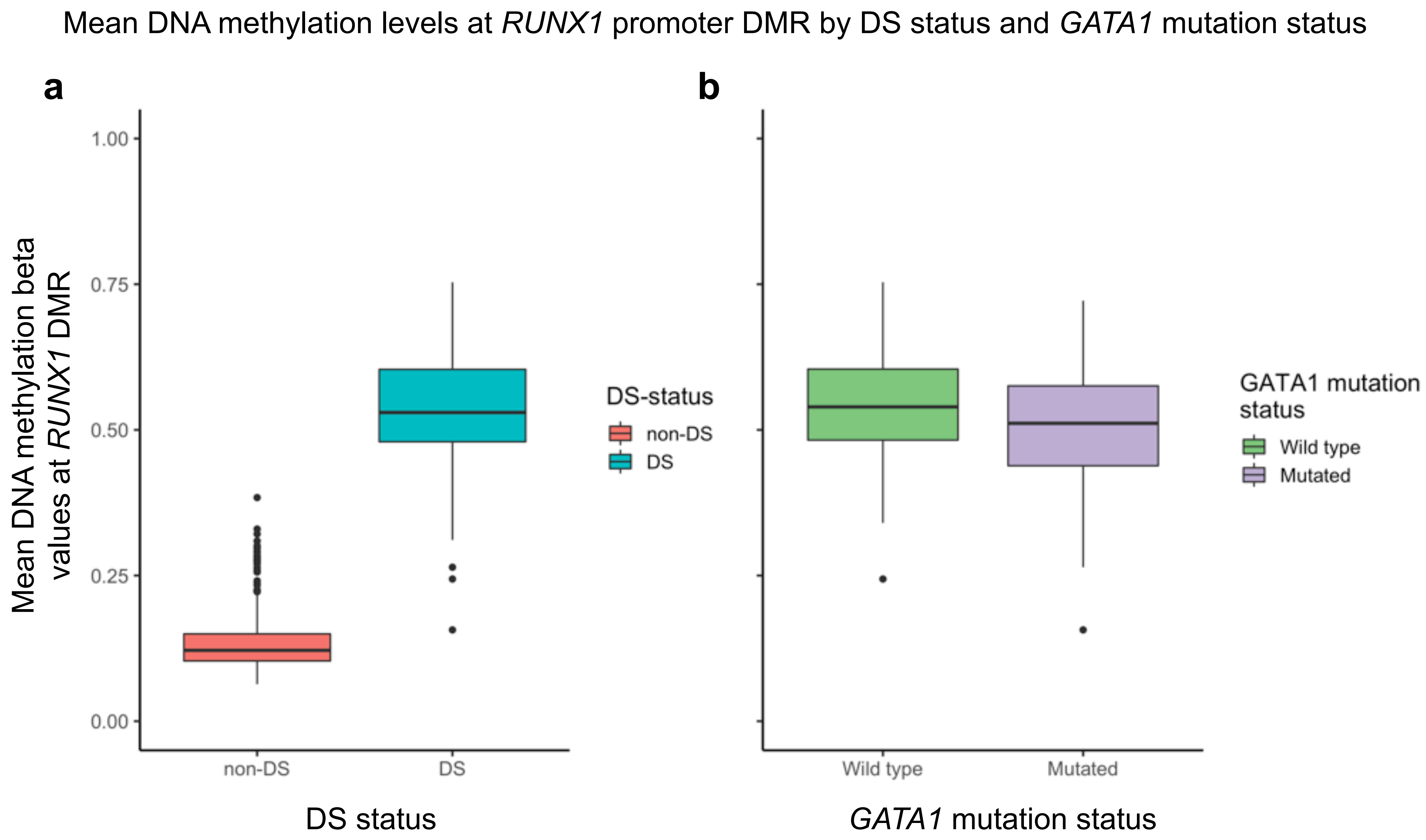
Mean DNA methylation levels at RUNX1 promoter DMR by DS status and GATA1 mutation status
a
b
Mean DNA methylation beta values at RUNX1 DMR
1.00
0.75
0.50
0.25
0.00
non-DS
DS
DS status
DS-status
non-DS
DS
Wild type
Mutated
GATA1 mutation status
GATA1 mutation status
Wild type
Mutated

Supplementary Figure 13 – *FLI1* promoter DMR mean DNA methylation levels in newborns with and without Down syndrome, and by *GATA1* mutation status. Boxplots displaying the mean DNA methylation beta values across 19 CpGs in a *FLI1* promoter DMR: (**a**) among non-DS newborns (N=439) versus DS newborns (N=196), and (**b**) among *GATA1* mutation-positive DS newborns (N=30) versus mutation wildtype (wt) newborns (N=154). In the boxplots, the center line represents the median, the upper and lower bounds of the box represent the interquartile range (IQR, the range between the 25th and 75th percentiles), and whiskers extend to the highest and lowest values within 1.5 times the IQR.

Figure S13

Mean DNA methylation levels at *FLI1* promoter DMR by DS status and *GATA1* mutation status

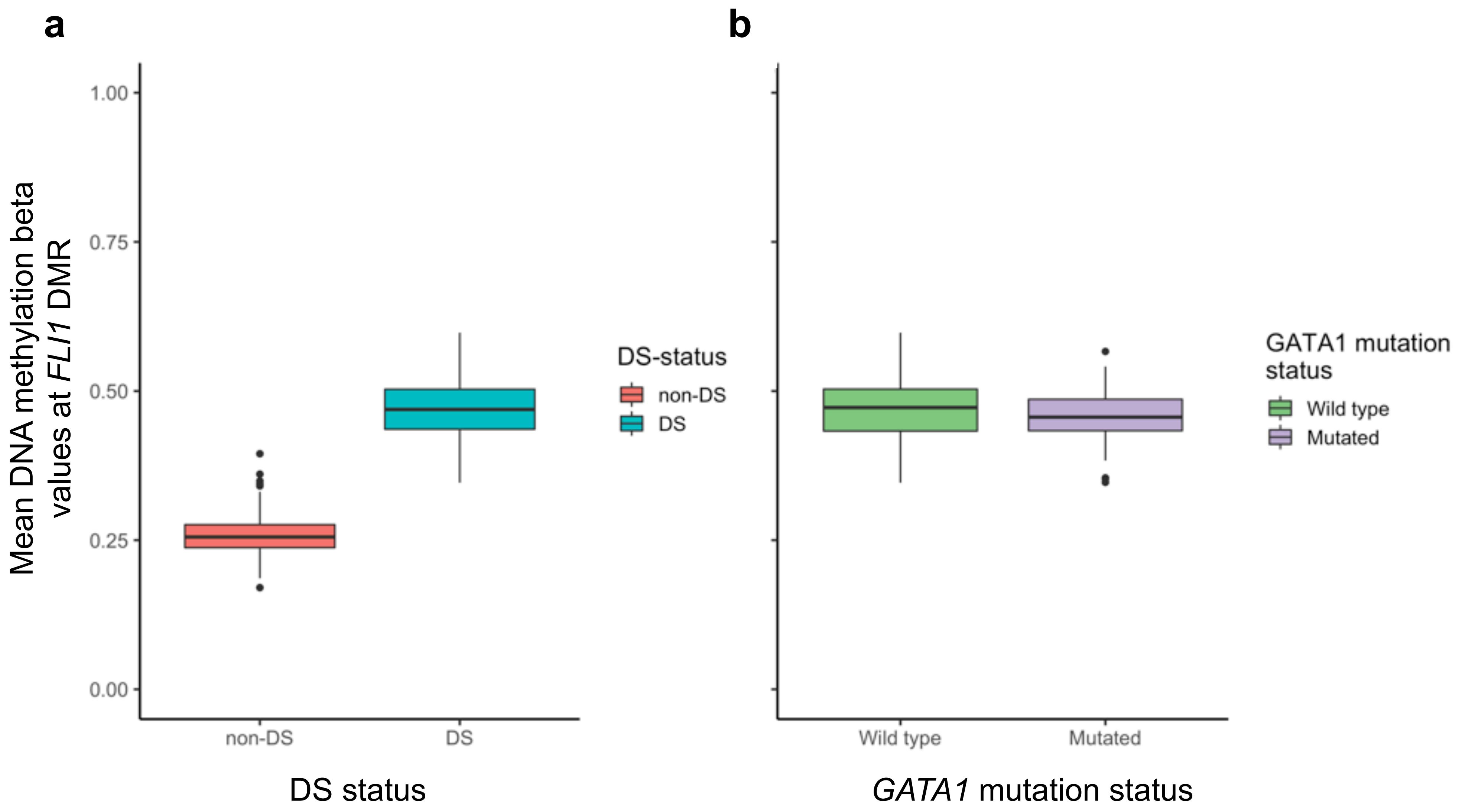

Supplementary Figure 14 – Correlation between differential methylation at Down syndrome-associated DMRs in gene body, intergenic regions, and on chromosome 21 in newborn dried bloodspots and differential gene expression in fetal liver CD34+ cells in DS and non-DS samples. Bar charts displaying the number of hypomethylated and hypermethylated DS-associated DMRs along with the direction of differential expression of corresponding genes in RNA-sequencing of DS (N=3) versus non-DS (N=3) FL CD34+ cells. Panel **a** displays results for DMRs on all chromosomes overlapping the gene body. Panel **b** displays results for DMRs on all chromosome overlapping intergenic regions, for which differential expression was compared for the nearest gene as classified by DMRcate. Results are also displayed for DMRs only on chromosome 21 and overlapping promoters/enhancers (**c**), gene body (**d**), and intergenic regions (**e**). P-values were calculated by two-sided Fisher's exact test.

Figure S14

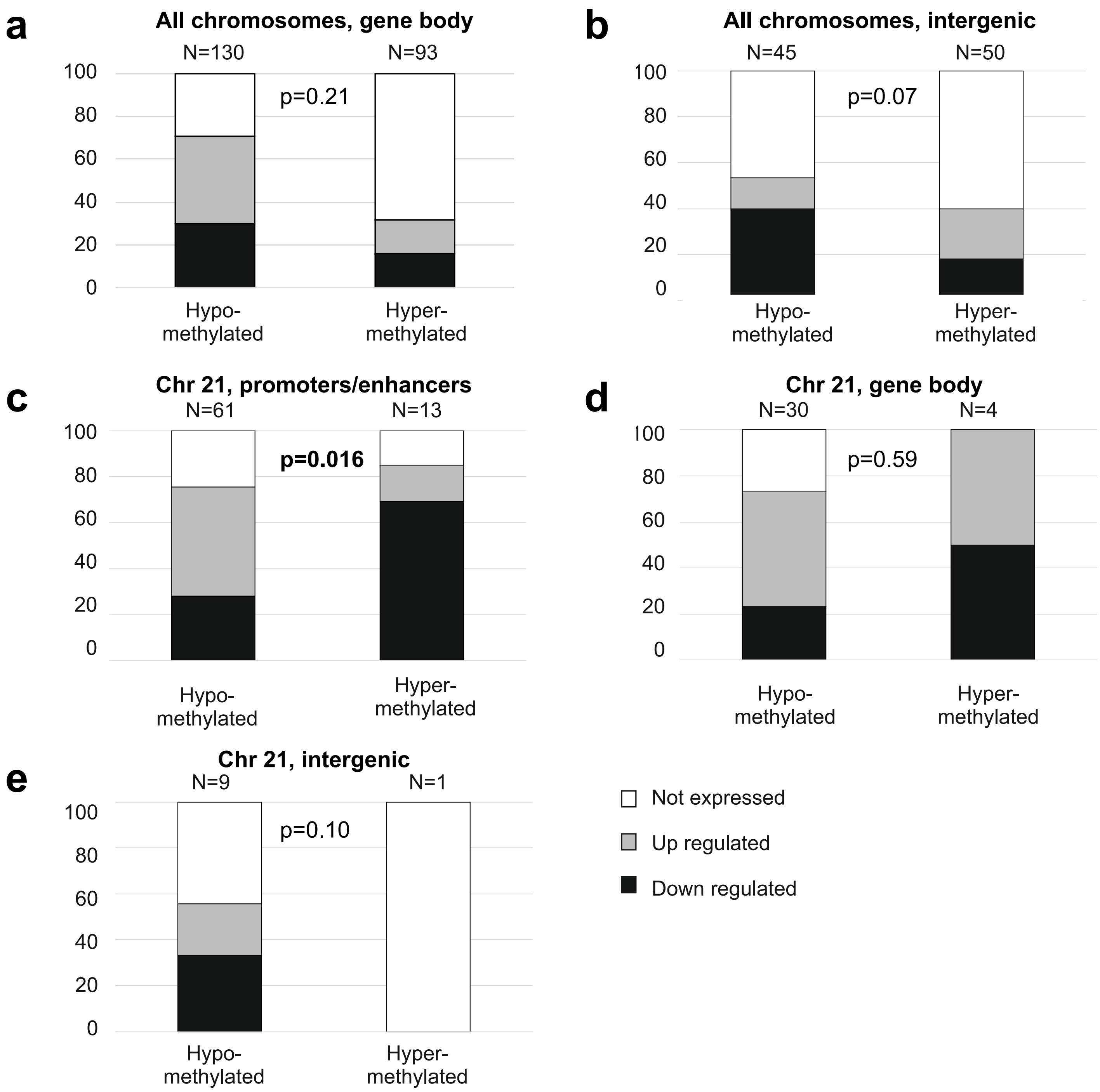
a
All chromosomes, gene body
N=130
N=93
100
80
60
40
20
0
p=0.21
Hypo-
methylated
Hyper-
methylated
b
All chromosomes, intergenic
N=45
N=50
p=0.07
Hypo-
methylated
Hyper-
methylated
c
Chr 21, promoters/enhancers
N=61
N=13
p=0.016
Hypo-
methylated
Hyper-
methylated
d
Chr 21, gene body
N=30
N=4
p=0.59
Hypo-
methylated
Hyper-
methylated
e
Chr 21, intergenic
N=9
N=1
p=0.10
Hypo-
methylated
Hyper-
methylated
Not expressed
Up regulated
Down regulated
