# Supplementary material for: The genome-wide impact of trisomy 21 on DNA methylation and its implications for hematopoiesis
Source: Nat Commun. 2021 Feb 5;12:821. doi: 10.1038/s41467-021-21064-z (PMC7865055; doi:10.1038/s41467-021-21064-z)
Supplement: Supplementary file 3 — Reporting Summary [file 41467_2021_21064_MOESM3_ESM.pdf]

nature research

Corresponding author(s): Adam J. de Smith (adam.desmith@med.usc.edu)

Last updated by author(s): 12/08/2020

Reporting Summary

Nature Research wishes to improve the reproducibility of the work that we publish. This form provides structure for consistency and transparency in reporting. For further information on Nature Research policies, see our Editorial Policies and the Editorial Policy Checklist.

Statistics

For all statistical analyses, confirm that the following items are present in the figure legend, table legend, main text, or Methods section.

n/a	Confirmed	
☐	☒	The exact sample size (*n*) for each experimental group/condition, given as a discrete number and unit of measurement
☒	☐	A statement on whether measurements were taken from distinct samples or whether the same sample was measured repeatedly
☐	☒	The statistical test(s) used AND whether they are one- or two-sided<br>*Only common tests should be described solely by name; describe more complex techniques in the Methods section.*
☐	☒	A description of all covariates tested
☐	☒	A description of any assumptions or corrections, such as tests of normality and adjustment for multiple comparisons
☐	☒	A full description of the statistical parameters including central tendency (e.g. means) or other basic estimates (e.g. regression coefficient) AND variation (e.g. standard deviation) or associated estimates of uncertainty (e.g. confidence intervals)
☐	☒	For null hypothesis testing, the test statistic (e.g. F, t, r) with confidence intervals, effect sizes, degrees of freedom and P value noted<br>*Give P values as exact values whenever suitable.*
☒	☐	For Bayesian analysis, information on the choice of priors and Markov chain Monte Carlo settings
☒	☐	For hierarchical and complex designs, identification of the appropriate level for tests and full reporting of outcomes
☐	☒	Estimates of effect sizes (e.g. Cohen's d, Pearson's r), indicating how they were calculated

Our web collection on statistics for biologists contains articles on many of the points above.

Software and code

Policy information about availability of computer code

Data collection	DNA methylation data were collected from Illumina Infinium MethylationEPIC Beadchip genome-wide DNA methylation arrays, run on the Illumina iScan system and processed in Genome Studio software. Targeted GATA1 sequencing data were obtained using an Illumina MiSeq system. RNA sequencing data were obtained from an Illumina HiSeq2500.
Data analysis	Software used included: R version 3.6.0, GLINT v1.0.4, VarScan2 v2.4.4, STAR v2.7, Integrative Genomics Viewer v2.7.<br>The following R packages were used: minfi v1.36, SeSAMe v1.7.10, conumee v1.24, impute v1.56, Rtsne v0.15, ComplexHeatmap v2.6.2, IlluminaHumanMethylationEPICanno.ilm10b4.hg19 v0.6.0, FlowSorted.Blood.EPIC v1.2.0, FlowSorted.CordBloodCombined.450k v1.2.1, DMRcate v2.4.0, combp, coMET v1.22, methylGSA v1.8.0, Subread v2.0.0, and DESeq2 v1.30.<br>Quality control of RNA sequencing data was performed using the FastQC v0.11.9 package (http://www.bioinformatics.babraham.ac.uk/projects/fastqc). Adapter trimming was performed using Trim Galore (https://github.com/FelixKrueger/TrimGalore).

For manuscripts utilizing custom algorithms or software that are central to the research but not yet described in published literature, software must be made available to editors and reviewers. We strongly encourage code deposition in a community repository (e.g. GitHub). See the Nature Research guidelines for submitting code & software for further information.

Data

Policy information about availability of data

All manuscripts must include a data availability statement. This statement should provide the following information, where applicable:

- Accession codes, unique identifiers, or web links for publicly available datasets
- A list of figures that have associated raw data
- A description of any restrictions on data availability

ENCODE TF binding site dataset wgEncodeRegTfbsClusteredV3.bed.gz was downloaded from the UCSC Genome Browser (https://hgdownload.cse.ucsc.edu/

goldenpath/hg19/encodeDCC/wgEncodeRegTfbsClustered/). Histone modification data were downloaded for primary hematopoietic stem cells (cell line E035, CD34 primary cells) from the Roadmap Epigenomics Mapping Consortium database (https://egg2.wustl.edu/roadmap/data/byFileType/peaks/). ENCODE DNase I hypersensitive sites data are available at http://hgdownload.cse.ucsc.edu/goldenpath/hg19/encodeDCC/wgEncodeRegDnaseClustered/. NHGRI-EBI GWAS Catalog data are available at https://www.ebi.ac.uk/gwas/docs/file-downloads. FLI1 transcript variant data were downloaded from the BLUEPRINT Consortium Blood Atlas (https://blueprint.haem.cam.ac.uk/mRNA/). This study used biospecimens from the California Biobank Program. Any uploading of genomic data (including genome-wide DNA methylation data) and/or sharing of these biospecimens or individual data derived from these biospecimens has been determined to violate the statutory scheme of the California Health and Safety Code Sections 124980(j), 124991(b), (g), (h), and 103850 (a) and (d), which protect the confidential nature of biospecimens and individual data derived from biospecimens. The individual-level data derived from these biospecimens and that support the findings of this study are available from the corresponding author upon request, and with permission from the California Biobank Program. RNA-seq data from DS and non-DS FL CD34+ cells have been deposited at the Gene Expression Omnibus (GEO) with accession code: GSE160637 (https://www.ncbi.nlm.nih.gov/geo/query/acc.cgi?acc=GSE160637). Remaining source data are provided with this paper.

Field-specific reporting

Please select the one below that is the best fit for your research. If you are not sure, read the appropriate sections before making your selection.

☒ Life sciences ☐ Behavioural & social sciences ☐ Ecological, evolutionary & environmental sciences

For a reference copy of the document with all sections, see nature.com/documents/nr-reporting-summary-flat.pdf

Life sciences study design

All studies must disclose on these points even when the disclosure is negative.

Sample size	Sample size was determined by the number of newborn bloodspot samples available for DNA methylation profiling at the time of study inception, rather than being predetermined by power analysis. The sample size is much larger than any previously published study of DNA methylation in Down syndrome compared to non-Down syndrome individuals. Smaller studies have reported significant Down syndrome-associated methylation probes.
Data exclusions	We performed stringent quality control to exclude poor quality probes and samples from the epigenome-wide association study. Poorly performing DNA methylation probes were removed using P-value with out-of-band (OOB) array hybridization (pOOBAH) during the QC process. One subject was removed as their chromosome 21 copy number status conflicted with their original inclusion as a non-Down syndrome control. Using pre-established exclusion criteria for sample and probe missingness, we removed subjects with missingness >5% (2 DS and 2 non-DS), and subsequently removed CpG probes with missingness >5% (N=137,060).
Replication	Additional large epigenome-wide association studies of Down syndrome do not currently exist with sufficient sample sizes to provide robust replication analyses. We carried out several measures to verify the reproducibility of our findings, in particular stratification of subjects into Latinos and non-Latino whites to ensure that significant Down syndrome-associated probes and regions in the overall analysis had consistent associations and direction of effects in the ethnicity stratified EWAS. In addition, we replicated a majority of DS-associated probes and regions identified in the previous largest EWAS of DS (Bacalini et al. 2015).
Randomization	Bisulfite-converted DNA samples from Down syndrome (DS) and non-DS newborns were block-randomized to ensure equivalent distribution of sex and race/ethnicity on the Illumina Infinium MethylationEPIC Beadchip genome-wide DNA methylation arrays.
Blinding	Investigators were blinded to Down syndrome status during the DNA isolation, bisulfite conversion process, and DNA methylation array preparations and QC process. During analysis of DNA methylation data, Down syndrome status was not blinded so that case-control association testing could be performed.

Reporting for specific materials, systems and methods

We require information from authors about some types of materials, experimental systems and methods used in many studies. Here, indicate whether each material, system or method listed is relevant to your study. If you are not sure if a list item applies to your research, read the appropriate section before selecting a response.

Materials & experimental systems

n/a	Involved in the study	
☒	☐	Antibodies
☒	☐	Eukaryotic cell lines
☒	☐	Palaeontology and archaeology
☒	☐	Animals and other organisms
☐	☒	Human research participants
☒	☐	Clinical data
☒	☐	Dual use research of concern

Methods

n/a	Involved in the study	
☒	☐	ChIP-seq
☐	☒	Flow cytometry
☒	☐	MRI-based neuroimaging

Human research participants

Policy information about studies involving human research participants

Population characteristics	All subjects included in the EWAS were newborns with Down syndrome (N=198) or without Down syndrome (N=442). The median age of blood draw for newborn dried bloodspot collection was 1.75 days for newborns with Down syndrome and 1.13 days for newborns without Down syndrome. Numbers of males and females were approximately even. Individuals self-identified as Latino (N=357) or non-Latino white (N=178), and the remainder as African American (N=34), Asian/Pacific Islander (N=55), or other (N=11)
Recruitment	For the EWAS, newborns with Down syndrome were identified by the California Biobank Program via linkage between the California Department of Public Health Genetic Disease Screening Program and California Cancer Registry (to exclude subjects that developed cancer during childhood) (PMID: 31350265). Newborns without Down syndrome were identified by the California Biobank Program (PMID: 30923090).<br>For gene expression analyses, second trimester fetal liver (FL) samples from DS subjects were collected during elective surgical termination of pregnancy and processed immediately. Donated fetal tissue was also provided by the Human Developmental Biology Resource (HDBR, www.hdbr.org) regulated by the UK Human Tissue Authority (HTA, www.hta.gov.uk).<br>There were no biases in the selection process that likely impacted results.
Ethics oversight	This study was approved by Institutional Review Boards at the California Health and Human Services Agency, University of Southern California, and University of California Berkeley, and by Hammersmith and Queen Charlotte's Hospitals Research Ethics Committee (ref 04/Q0406/145).

Note that full information on the approval of the study protocol must also be provided in the manuscript.

Flow Cytometry

Plots

Confirm that:

- [ ] The axis labels state the marker and fluorochrome used (e.g. CD4-FITC).
- [ ] The axis scales are clearly visible. Include numbers along axes only for bottom left plot of group (a 'group' is an analysis of identical markers).
- [ ] All plots are contour plots with outliers or pseudocolor plots.
- [ ] A numerical value for number of cells or percentage (with statistics) is provided.

Methodology

Sample preparation	Single cells from fetal liver common myeloid progenitors (CMP:Lin-CD34+CD38+CD45RA-CD123+) and megakaryocyte-erythroid progenitors (MEP:Lin-CD34+CD38+CD45RA-CD123-) from 3 DS and 3 non-DS subjects were index sorted into a 96-well plate containing pre-amplification mix.
Instrument	Samples were FACS-sorted using BD Fusion instruments.
Software	Data were analyzed using FlowJo software.
Cell population abundance	*Describe the abundance of the relevant cell populations within post-sort fractions, providing details on the purity of the samples and how it was determined.*
Gating strategy	Gating strategy for flow cytometry experiments already published and referenced in our manuscript: O'Byrne et al. (2019) Blood, PMID:31383639, https://doi.org/10.1182/blood.2019001289

- [ ] Tick this box to confirm that a figure exemplifying the gating strategy is provided in the Supplementary Information.
