# Supplementary material for: The genome-wide impact of trisomy 21 on DNA methylation and its implications for hematopoiesis
Source: Nat Commun. 2021 Feb 5;12:821. doi: 10.1038/s41467-021-21064-z (PMC7865055; doi:10.1038/s41467-021-21064-z)
Supplement: Supplementary file 4 — Description of Additional Supplementary Files [file 41467_2021_21064_MOESM4_ESM.pdf]

Description of Additional Supplementary Files

Supplementary Data 1 – Somatic GATA1 mutations detected across 184 newborns with Down syndrome by targeted sequencing.

Supplementary Data 2 – a: Differentially methylated autosomal CpG probes associated with Down syndrome (DS) at epigenome-wide significance; b: Differentially methylated CpG probes on chromosome X associated with Down syndrome at epigenome-wide significance, stratified by sex.

Supplementary Data 3 – Enrichment of Down syndrome-associated CpG probes genome-wide and on Hsa21 in: a) genomic locations, b) histone modification markers in hematopoietic stem cells, c) predicted enhancer regions, d) Transcription factor binding sites in K562 cells, and e) DNase I hypersensitive sites (DHS) in ENCODE. P-values were calculated by twosided Fisher's exact tests.

Supplementary Data 4 – Gene pathway enrichment analysis results for genes overlapped by Down syndrome-associated CpGs, with assessment of (a) Gene Ontology (GO) and (b) Kyoto Encyclopedia of Genes and Genomes (KEGG) pathways.

Supplementary Data 5 – a: Replication of differentially methylated CpG probes previously associated with Down syndrome; b: Replication of differentially methylated regions (DMRs) previously associated with Down syndrome.

Supplementary Data 6 – a: Differentially methylated CpG probes associated with GATA1 mutation status in newborns with Down syndrome; b: Differentially methylated regions (DMRs) associated with GATA1 mutation status in newborns with Down syndrome.

Supplementary Data 7 – Differentially methylated regions (DMRs) associated with Down syndrome (DS), identified by both DMRcate and comb-p methods: a) overall, b) in Latinos, c) in non-Latino whites, d) excluding high nRBC DS newborns, and e) excluding GATA1 mutationpositive DS newborns.

Supplementary Data 8 – NHGRI-EBI Catalog of published genome-wide association studies (GWAS Catalog) SNPs overlapping Down syndromeassociated DMRs, and the SNP-associated phenotypes (a) and metadata (b).

Supplementary Data 9 – Differentially expressed genes in Down syndrome fetal liver (N=3) versus non-Down syndrome fetal liver (N=3) CD34+ cells, assessed using DESeq2.
