# Supplementary material for: The genome-wide impact of trisomy 21 on DNA methylation and its implications for hematopoiesis
Source: Nat Commun. 2021 Feb 5;12:821. doi: 10.1038/s41467-021-21064-z (PMC7865055; doi:10.1038/s41467-021-21064-z)
Supplement: Supplementary file 2 — Peer Review File [file 41467_2021_21064_MOESM2_ESM.pdf]

Reviewer #1 (Remarks to the Author):

In this manuscript, Nuskens et al performed an epigenome-wide association study on neonatal bloodspots from DS and non-DS individuals. Unsupervised clustering on DNA methylation data separated DS and non-DS newborn, and this result was in part trained by the large differences in peripheral blood cell composition between the two groups. GATA1 mutation status had a lower effect on epigenomic profiles. The search for differentially methylated positions and regions identified several loci with a potential relevance in DS pathology, including d RUNX1 and FLI1.
This is an excellent piece of work, performed on a large DS cohort. The analytical methods that have been applied are appropriate and the discussion is supported by the results.
I have only minor comments:
- in the abstract, the results from unsupervised analysis and on peripheral blood cell composition analysis are not mentioned, while they have large space in the results section. I would suggest to mention these results also in the abstract.
- two different methods for cell composition deconvolution have been used (FlowSorted.CordBloodCombined.450k and ReFACTor). I think that the differences between the two approaches are not clearly explained and are confusing for a general reader. You should comment this point at the end of the "Deconvolution of blood cell proportions" section.

Reviewer #2 (Remarks to the Author):

Your manuscript describes and EWAS comparison of neonatal blood DNA from neonates with and without Down syndrome (DS). You claim that the differential methylation you find explains some of the blood-related phenotypes seen in those with DS which s plausible and a valuable addition to the field. Your finding will be of interest to the general epigenetics and medical community too. The statistical analyses you use is appropriate , although I have some comments for you to address in this area. Overall, this is the kind of paper that I as an epigenetics researcher would find very interesting and medically relevant. I trust you will be able to address the general and specific comments I list below.

General comments
Information included in the legend of figures such as 2A is a little excessive and comes as a surprise to the reader who is not provided with rationale of methods detail prior to the figures. Can the authors better prepare the reader?
The Discussion alludes to comparing probes from Hsa21 and all other probes, but this should be a more explicit part of the study for obvious regions such as issues of dosage compensation.
The authors talk about multi-ethnicity but should provide details in Results section manuscript of the ethnicities included

Specific comments
Results, lines 96-97: please state the platform used here and the number of CpGs analysed
Results: As differences were found between the two groups in gestational age and birth weight, can the authors justify why they didn't include these in the regression model?
In Figure 1 you mention that CpG probes from chromosomes X, Y, and 21 were not included in your analysis but in the paragraph starting on line 149 you talk about Hsa21 probes. This is confusing; can you please make it plain which chromosomes were excluded from which analysis ?
Lines 236-238: the word 'differences' should be applied to expression as well as methylation
Discussion, line 344-5: I am not convinced that of the authors' assertion that maternal diet is implicated in the development of GATA1 mutations. Can the authors please expand on this?
Discussion: the authors mention that "our EWAS was adjusted for sex and principal components". This is a very important issue that should have been mentioned earlier.
Discussion, line 352-3: I don't follow the authors' rationale for suggesting that cell type deconvolution

algorithms should have been developed in non-euploid individuals. Can they please explain or delete this ?
Discussion: can the authors please discuss the dilemma in adjusting for cell type proportions versus not adjusting, and letting the results reflect the true differences in blood cell heterogeneity as phenotypic dimension of DS?

Jeffrey M Craig

<u>Response to Reviewer Comments:</u>

We are extremely grateful to the reviewers for their positive feedback and constructive criticisms, and for their thorough reviews of our manuscript. Please find our point-by-point responses to the reviewer comments below:

Reviewer #1 (Remarks to the Author):

In this manuscript, Muskens et al performed an epigenome-wide association study on neonatal bloodspots from DS and non-DS individuals. Unsupervised clustering on DNA methylation data separated DS and non-DS newborn, and this result was in part trained by the large differences in peripheral blood cell composition between the two groups. GATA1 mutation status had a lower effect on epigenomic profiles. The search for differentially methylated positions and regions identified several loci with a potential relevance in DS pathology, including d RUNX1 and FLI1.
This is an excellent piece of work, performed on a large DS cohort. The analytical methods that have been applied are appropriate and the discussion is supported by the results.

~ We thank the reviewer for their positive feedback.

I have only minor comments:

- in the abstract, the results from unsupervised analysis and on peripheral blood cell composition analysis are not mentioned, while they have large space in the results section. I would suggest to mention these results also in the abstract.

~ Due to the 150 word limit of abstracts in Nature Communications articles, we decided to focus more on the novel results in our study and did not have space to discuss other results, such as the differences in blood cell proportions between DS and non-DS individuals, some of which have been previously reported. However, we have now amended the Abstract to state that the EWAS was performed, "<u>adjusting for cell-type heterogeneity</u>" as we realize this is important to highlight.

- two different methods for cell composition deconvolution have been used (FlowSorted.CordBloodCombined.450k and ReFACTor). I think that the differences between the two approaches are not clearly explained and are confusing for a general reader. You should comment this point at the end of the "Deconvolution of blood cell proportions" section.

~ We thank the reviewer for highlighting this, and have attempted to address any confusion in the Results section, "Deconvolution of blood cell proportions." Firstly, on line 137 we have added that we used "<u>reference-based</u> cell-type deconvolution…" to assess the difference in blood cell proportions between DS and non-DS newborns. Second, at the end of this section (starting line 158) we have added the following sentence, which we hope will clarify the difference between the method used to estimate differences in blood cell proportions and the method we subsequently used to generate components to include as covariates in the EWAS of DS: "<u>Rather than adjusting for the blood cell proportions estimated from our reference-based deconvolution described above, we opted to include components calculated using the reference-free, sparse principal component analysis algorithm ReFACTor (see Methods) as covariates in our EWAS models.</u>"

We also added the term “Reference-based deconvolution” in the Methods section at line 433.

Reviewer #2 (Remarks to the Author):

Your manuscript describes and EWAS comparison of neonatal blood DNA from neonates with and without Down syndrome (DS). You claim that the differential methylation you find explains some of the blood-related phenotypes seen in those with DS which s plausible and a valuable addition to the field. Your finding will be of interest to the general epigenetics and medical community too. The statistical analyses you use is appropriate, although I have some comments for you to address in this area. Overall, this is the kind of paper that I as an epigenetics researcher would find very interesting and medically relevant. I trust you will be able to address the general and specific comments I list below.

~ We thank the reviewer for their positive feedback, and hope that we have addressed their general and specific comments below sufficiently.

General comments

Information included in the legend of figures such as 2A is a little excessive and comes as a surprise to the reader who is not provided with rationale of methods detail prior to the figures. Can the authors better prepare the reader?

~ We thank the reviewer for highlighting this point. We were somewhat limited by the word limit and the positioning of the Methods section at the end of the manuscript as per journal requirements, but we have attempted to better prepare the reader for some of the figure content as follows:

- *Figure 1: we have added a sentence in the Results at lines 111-113 that the PCA and tSNE plots were “generated from genome-wide DNA methylation data, excluding CpG probes on sex chromosomes and Hsa21 and CpGs overlapping single nucleotide polymorphisms (SNPs) with minor allele frequency (MAF) >0.05” and removed some of these details from the Figure 1 legend.*
- *Figure 2: we have added in the Results at lines 116-117 that unsupervised clustering was performed for “the top 2000 most variable CpGs genome-wide”, and at lines 120-121 that “Differences in blood cell proportions inferred from genome-wide DNA methylation data were seen between the three groups, as described in detail below.” We have similarly removed some of the details from the Figure 2 legend, including those obvious from the figure key.*
- *Figure 3: we have removed some details from the legend, including information that was already present in the Methods and Results sections such as the association test used and number of CpGs included.*
- *We have also cut some text from the legends of Figures 4 and 6.*

The Discussion alludes to comparing probes from Hsa21 and all other probes, but this should be a more explicit part of the study for obvious regions such as issues of dosage compensation.

~ Several prior studies reported that Down syndrome is associated with hypomethylation of Hsa21, and we did report in the Results section that, “The pattern of DNA methylation was distinctly different for CpGs on Hsa21; the vast majority of differentially methylated CpGs (64/79; 81.0%) were hypomethylated, whereas on

other chromosomes the proportions of hypomethylated CpGs were on average much lower (median:43.5%, range:16.0-80.0%).”

We have now expanded this analysis to look at all of the CpGs included in the EWAS, not only limited to the epigenome-wide significant ones, and we found that a significantly higher proportion of CpGs on Hsa21 were hypomethylated compared with CpGs across the other autosomes. We include a new Supplementary Table 5 including these results overall and by CpG island location, and have added the following to the Results on lines 173-177: “In addition, when considering all CpGs included in the EWAS, a significantly higher proportion of probes on Hsa21 were hypomethylated (4425/7351; 60.2%) compared with probes on all other autosomes combined (356,222/644,421, 55.3%; P<0.0001, Chi-squared test), which was particularly the case in shores and shelves but not in CpG islands themselves (Supplementary Table 5).”

The authors talk about multi-ethnicity but should provide details in Results section manuscript of the ethnicities included

~ We thank the reviewer for this suggestion, and have now added the following sentence to the first section of the Results at lines 101-102, “Study characteristics of the 635 newborns (N=357 Latinos, 178 non-Latino whites, 55 Asians, 34 non-Latino blacks, and 11 Other) are presented in ***Table 1****.”*

Specific comments

Results, lines 96-97: please state the platform used here and the number of CpGs analysed

~ We have now added information on the Illumina array used and the number of CpGs included in our analyses.

Results: As differences were found between the two groups in gestational age and birth weight, can the authors justify why they didn’t include these in the regression model?

~ The reviewer raises a great point. We did not include these birth variables in the overall EWAS of Down syndrome because gestational age and birthweight data were not available for all DS and non-DS newborns. But to address this point, we have now repeated the EWAS of DS in a subset of newborns with available birth variable data (176/196 DS and 416/435 non-DS) and adjusting for gestational age and birthweight, and found that the results remained largely consistent with our overall analysis.

For example, there was a high correlation between the beta values generated for each CpG from the EWAS with or without adjustment for birthweight and gestational age, as shown in the scatterplot below:

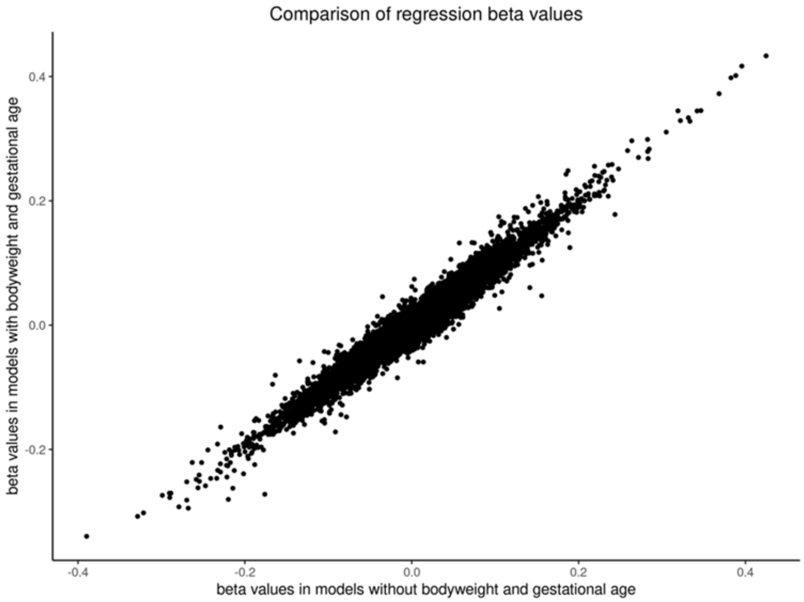

Pearson's R = 0.974

We have now added two columns to Supplementary Table 4 (EWAS significant CpGs) that include the beta values and P-values of the 652 epigenome-wide significant CpGs from an EWAS model adjusting for both birthweight and gestational age.

As a snapshot, below is a table showing the results for the top 20 CpGs with and without this adjustment. On average, the beta values for these CpGs changed less than 5%.

topCpG	Gene	Beta_adjusted_bweight_gestage	Pvalue_adjusted_bweight_gestage	OriginalBeta	OriginalP
cg07741821	KIAA0087 (lncRNA)	-0.2702837	4.94E-34	-0.2886799	1.26E-39
cg02993069	SH3D21	0.19031343	7.07E-23	0.19754932	1.14E-25
cg12477880	RUNX1	0.39791923	2.18E-26	0.3826307	2.32E-25
cg08882472	DST	0.15694662	4.49E-23	0.15737323	6.14E-25
cg24942416	VSIG2	-0.1645007	1.19E-20	-0.1750691	7.33E-24
cg07841633	intergenic	-0.3076814	1.77E-19	-0.3285122	4.21E-23
cg00994804	RUNX1	0.40149229	2.42E-22	0.38840898	9.36E-22
cg11218872	intergenic	-0.1118466	3.73E-19	-0.1208576	1.05E-21
cg02451831	KIAA0087 (lncRNA)	-0.1564687	1.25E-19	-0.1585435	2.67E-21
cg13382072	KLF16	0.18236035	1.60E-20	0.17988851	4.07E-21
cg24020235	intergenic	0.19489456	9.47E-21	0.1875985	7.30E-21
cg17239923	FLI1	0.22557606	1.37E-18	0.23218845	1.21E-20
cg19030331	OLFML1	0.16328652	1.44E-18	0.16510215	6.05E-20
cg03142697	RUNX1	0.41674595	2.93E-20	0.39556858	2.15E-19
cg24999883	SETD3	-0.0714893	4.55E-19	-0.0671554	2.85E-19
cg12679760	SH3D21	0.17423668	1.99E-16	0.18286939	9.10E-19
cg23719650	intergenic	-0.0977138	3.24E-16	-0.1070741	1.11E-18
cg11972401	NOL10	0.06175813	2.85E-18	0.05787608	2.10E-18
cg23565347	CELF3	-0.0944556	2.09E-17	-0.0920343	2.15E-18
cg19765472	FLI1	0.19988277	2.56E-16	0.20671829	2.42E-18

*Finally, we have added the following sentence to the Results on lines 194-196, "Additionally, in a subset of newborns with available birthweight and gestational age information (176 DS, 416 non-DS), we repeated the EWAS adjusting for these birth variables but again results were not substantially altered (**Supplementary Table 4**)."*

In Figure 1 you mention that CpG probes from chromosomes X, Y, and 21 were not included in your analysis but in the paragraph starting on line 149 you talk about Hsa21 probes. This is confusing; can you please make it plain which chromosomes were excluded from which analysis ?

~ We thank the reviewer for highlighting this confusion, and we have added the following amendments that we hope will help to clarify which chromosomes were excluded from which analysis:

- *In the Results on line 98, when we first state the number of CpGs analyzed we note that these were "on autosomes"*
- *At lines 111-112, we have added to the sentence first describing PCA and t-SNE plots that these were "generated from genome-wide DNA methylation data, excluding CpG probes on sex chromosomes and Hsa21"*
- *At lines 116-117, we have added that the hierarchical clustering was performed using "the top 2000 most variable CpGs genome-wide (excluding chromosomes 21, X, and Y)"*
- *At lines 165-166, we have added that "we next assessed differential methylation of CpGs on autosomal chromosomes including Hsa21"*

Lines 236-238: the word 'differences' should be applied to expression as well as methylation

~ This has been amended accordingly.

Discussion, line 344-5: I am not convinced that of the authors' assertion that maternal diet is implicated in the development of GATA1 mutations. Can the authors please expand on this?

~ In retrospect this assertion was too speculative. The DMR at VTRNA2-1, which we found to be associated with GATA1 *mutation status in newborns with Down syndrome, has previously been associated with maternal periconceptional nutrition (PMID: 26062908). But given a lack of any information on maternal diet in our study, we have now revised this sentence at lines 370-371 as follows, "Our EWAS of GATA1 mutations in DS revealed a DMR overlapping VTRNA2-1, a metastable epiallele at which DNA methylation levels were previously associated with the periconceptional environment,[54] suggesting a potential environmental role in the development of GATA1 mutations."*

Discussion: the authors mention that "our EWAS was adjusted for sex and principal components". This is a very important issue that should have been mentioned earlier.

~ We thank the reviewer for highlighting this important point. Although we mention later on in the Methods section that we adjusted for sex and principal components (PCs) in our EWAS models, this was not described earlier in the Results. We have now added in the Results sections "Epigenome-wide significant CpGs associated with Down syndrome" and "Differentially methylated regions (DMRs) associated with Down syndrome" that we adjusted our models for "sex, and ancestry-informative PCs."

Discussion, line 352-3: I don’t follow the authors’ rationale for suggesting that cell type deconvolution algorithms should have been developed in non-euploid individuals. Can they please explain or delete this?

> *~ On the reviewer’s recommendation we have now deleted the following from line 380, “future studies should develop reference datasets in DS populations.” We have also now become aware of results reported in the Bacalini et al. (2014) study that demonstrated that using cell type deconvolution faithfully recapitulated actual white blood cell count measures in their set of 29 DS individuals. Along with our findings, this further supports that current cell type deconvolution methods are appropriate for inferring cell type proportions from DNA methylation array data in DS individuals.*

Discussion: can the authors please discuss the dilemma in adjusting for cell type proportions versus not adjusting, and letting the results reflect the true differences in blood cell heterogeneity as phenotypic dimension of DS?

> *~ The main motivation of this study was to identify the epigenetic effects of DS that may underlie DS-associated phenotypes. Given the highly significant differences in estimated blood cell proportions between the DS and non-DS newborns, we adjusted for cell type heterogeneity in the EWAS in order to remove as much “noise” as possible caused by blood cell heterogeneity, and thus improve our ability to detect the true epigenetic effects of trisomy 21 that may influence DS-associated phenotypes. We have now amended the following from line 382-388 of the Discussion: “Reference-free adjustment for cell type composition was performed in our EWAS<u>, given the highly significant differences in estimated blood cell proportions and to maximize our power to detect epigenetic changes associated with trisomy 21;</u> however, we cannot rule out that some of the DNA methylation changes associated with DS may reflect differences in peripheral blood cell composition between DS and non-DS newborns<u>, and future studies should explore the epigenetic effects of DS in sorted blood cells</u>.”*

Jeffrey M Craig

Reviewer #2 (Remarks to the Author):

Thank you for your detailed reply to my comments. I am satisfied that you have addressed them all.
